# Foreign Language Learning in Older Adults: Anatomical and Cognitive Markers of Vocabulary Learning Success

**DOI:** 10.3389/fnhum.2022.787413

**Published:** 2022-03-07

**Authors:** Manson Cheuk-Man Fong, Matthew King-Hang Ma, Jeremy Yin To Chui, Tammy Sheung Ting Law, Nga-Yan Hui, Alma Au, William Shiyuan Wang

**Affiliations:** ^1^Research Centre for Language, Cognition, and Neuroscience, Department of Chinese and Bilingual Studies, The Hong Kong Polytechnic University, Kowloon, Hong Kong SAR, China; ^2^Research Institute for Smart Ageing, The Hong Kong Polytechnic University, Kowloon, Hong Kong SAR, China; ^3^Department of Electronic Engineering, The Chinese University of Hong Kong, Hong Kong, Hong Kong SAR, China; ^4^Department of Applied Social Science, The Hong Kong Polytechnic University, Kowloon, Hong Kong SAR, China

**Keywords:** foreign language learning, vocabulary learning, structural MRI, FreeSurfer, pars orbitalis, caudal middle frontal cortex, semantic memory, episodic memory

## Abstract

In recent years, foreign language learning (FLL) has been proposed as a possible cognitive intervention for older adults. However, the brain network and cognitive functions underlying FLL has remained largely unconfirmed in older adults. In particular, older and younger adults have markedly different cognitive profile—while older adults tend to exhibit decline in most cognitive domains, their semantic memory usually remains intact. As such, older adults may engage the semantic functions to a larger extent than the other cognitive functions traditionally considered the most important (e.g., working memory capacity and phonological awareness). Using anatomical measurements and a cognitive test battery, the present study examined this hypothesis in twenty cognitively normal older adults (58–69 years old), who participated in a two-month Italian learning programme. Results showed that the immediate learning success and long-term retention of Italian vocabularies were most consistently predicted by the anatomical measures of the left pars orbitalis and left caudal middle frontal cortex, which are implicated in semantic and episodic memory functions. Convergent evidence was also found based on the pattern of cognitive associations. Our results are consistent with a prominent role of semantic and episodic memory functions in vocabulary learning in older learners.

## 1. Introduction

### 1.1. Foreign Language Learning in Older Adults

In foreign language learning (FLL) research, older adults have been an understudied population (Mackey and Sachs, 2012), which may be caused by the increased difficulty in picking up a new language after the “critical period” (Lenneberg, 1967; Hartshorne et al., 2018, see also Wang, 2018). However, there has been a recent change in attitude, due in part to a series of pioneering studies on the effect of lifelong bilingualism on brain structures and cognitive functions (Bialystok et al., 2007; Luk et al., 2011; Costa and Sebastián-Gallés, 2014; Olsen et al., 2015) and on the neuroplasticity induced by intensive FLL in younger adults (Wong and Perrachione, 2007; Mårtensson et al., 2012; Stein et al., 2012; Zatorre, 2013; Qi et al., 2019).

Relative to older monolinguals, older bilinguals were reported to have greater protection against dementia, better inhibition function, and improved executive control (Bialystok et al., 2007; van den Noort et al., 2019). The bilingual advantage has also been reported for other cognitive domains, e.g., episodic and verbal memory (Schroeder and Marian, 2012; Grant et al., 2014, but see Paap et al., 2015). Neurally, the correlates of bilingual experiences have been studied using many anatomical segmentation tools (Li et al., 2014; Hämäläinen et al., 2018; Maschio et al., 2019). Using a technique known as surface-based morphometry (SBM; Fischl and Dale, 2000; Winkler et al., 2010; Luders et al., 2012), early acquisition of two languages is found to be associated with larger surface area in the left pars opercularis of the inferior frontal gyrus (IFG) and right superior temporal gyrus (STG), while late acquisition is associated with increased mean curvature in the left STG (Hämäläinen et al., 2018). Bilingualism-induced neural differences have also been demonstrated using related anatomical measures such as sub-cortical volumes (DeLuca et al., 2019b) or gray matter density (Abutalebi et al., 2015), obtained by volume-based morphometry and voxel-based morphometry. For example, an expansion of the thalamus was observed for individuals with greater immersion in the second language (Pliatsikas et al., 2017; Deluca et al., 2019a).

As for learning-induced neuroplasticity in younger adults, five months of intensive FLL was sufficient to bring about increases in gray matter volume in the left IFG and left anterior temporal lobe (Stein et al., 2012). Intensive vocabulary learning in young interpreters was found to promote increases in cortical thickness across various regions in the left-lateralized language network, including left middle frontal gyrus, IFG, and STG, along with increased volume in the bilateral hippocampus (Mårtensson et al., 2012). Consistent with the classroom-based learning above, a recent experimental work based on either the picture–word association or virtual reality as the FLL paradigm revealed both structural and functional changes in younger adults, including increased cortical thickness over both the core language areas in the left hemisphere and many regions in the right hemisphere (Legault et al., 2019a). In another study, increased brain volume was found in the right hippocampus (Bellander et al., 2016).

Taken together, the findings on the bilingual advantage and learning-induced neuroplasticity have raised the possibility to use FLL as a non-pharmacological means for promoting successful aging in older adults (Antoniou et al., 2013; Antoniou and Wright, 2017; Ware et al., 2017; Klimova, 2018; Pfenninger and Polz, 2018; Klimova and Pikhart, 2020). Consistent with this conjecture, a few studies reported cognitive improvements in older learners after intensive FLL (Bak et al., 2016; Bubbico et al., 2019; Wong et al., 2019). For example, improvement in global cognition was found following six months of individual computer-based cognitively stimulating activities, including FLL and cognitive games (Wong et al., 2019). In another study, older learners also showed significant improvement in global cognition, along with increased resting-state functional connectivities (RSFCs) between posterior cingulate cortex and three right-hemispheric regions, including right IFG, right superior frontal gyrus, and left superior parietal lobule (Bubbico et al., 2019). This interesting finding suggests that the right-hemisphere homologs of the left-hemisphere language areas may provide “scaffolding” in the early phase of second language learning, before a right-to-left shift occurs (Yang et al., 2015; Qi and Legault, 2020). Despite the positive findings above, results have been mixed, with a few studies reporting no improvement for all cognitive skills examined (Ware et al., 2017; Berggren et al., 2020), some degree of enhancement that was however not significantly different from that of a control group (Valis et al., 2019), or neither learning-induced anatomical nor behavioral changes (Nilsson et al., 2021).

### 1.2. Neurocognitive Bases of Foreign Language Learning

To account for the neuroplastic changes or cognitive gains that could be driven by FLL in older adults, an understanding about the neurocognitive bases of FLL is essential, because the learning-induced changes in a brain region likely arise due to its increased recruitment over the learning programme (Wong et al., 2007). Toward this end, some prominent neuroanatomical models of language (e.g., Ullman, 2001a, 2004; Hickok and Poeppel, 2007) have provided the guiding theoretical framework. In the declarative–procedural model (DP model; Ullman, 2001a, 2004), the declarative memory (comprising semantic and episodic memory), as subserved by a cortical–hippocampal system (Mcclelland et al., 1995; Eichenbaum, 2000), is posited to be involved for learning words. In contrast, the procedural memory, as subserved by the insula and basal ganglia (especially the striatum), is recruited in phonological and rule-based grammar learning. Functional activation and connectivities studies have been central to the quest for the neurocognitive bases of FLL (e.g., Mestres-Missé et al., 2008; Abutalebi and Green, 2016; Bakker-Marshall et al., 2021, for a recent review, see Qi and Legault, 2020). However, one source of information that could not be understated is individual differences—the focus of the present article. Traditionally, they are sometimes considered as “noise” that may obscure the effect of interest. Nonetheless, they have proved reliable for linking brain functions and cognitive behavior (e.g., Kanai and Rees, 2011; Chiarello et al., 2013).

In FLL research, individual differences in brain functions are posited to put strong constraints on the learning success (Zatorre, 2013; Li and Grant, 2016). Previous behavioral and neural findings have suggested that phonological skills, such as phonological short-term memory (PSTM) and phonological awareness, are the determining factors of language learning success (Papagno et al., 1991; Baddeley et al., 1998; Koda, 1998; Linck et al., 2014; Gillon, 2017). Corroborating evidence has been found in both classroom-based and laboratory-based studies with younger learners, for phonemic learning (Golestani and Zatorre, 2009; Silbert et al., 2015; Fuhrmeister and Myers, 2021), lexical tone learning (Wong and Perrachione, 2007; Chandrasekaran et al., 2010), and artificial grammar learning (Yang and Li, 2012; Kepinska et al., 2017). In agreement with the view that PSTM plays a crucial role in FLL, functional connectivity or activation studies have reported an association between FLL success and regions important for phonological processing, e.g., left precentral gyrus (Veroude et al., 2010), left supramarginal gyrus (SMG; Veroude et al., 2010; Sliwinska et al., 2012), and left posterior STG (e.g., Wong et al., 2007). Also, consistent with the hypothesis that phonological awareness plays a critical role in language learning success, individual variation in learning outcomes can be predicted by individual variation in baseline sensitivity to non-lexical pitch patterns (Wong et al., 2007).

Of special interest to the present study are the anatomical associations with learning performance. For example, the success in linguistic pitch learning or direction discrimination were predicted by the volume of left Mescal's gyrus (Wong et al., 2008) and by the cortical thickness of the right hemispheric homolog of the Broca's area (Novén et al., 2019). In the study by Legault et al. (2019a), reviewed in the last section, the FLL performance was associated with the cortical thickness of right IFG in the picture–word group, and with the cortical thickness of right inferior parietal lobe in the VR group. The emphasis of phonological skills has extended to sub-cortical regions. Recent empirical works (Legault et al., 2019a) and theoretical papers (Abutalebi and Green, 2007; Li et al., 2014) have highlighted the roles of the striatum—comprising caudate nucleus and putamen—in phonological learning. For example, the caudate nucleus is involved in phonemic fluency (rather than semantic fluency; Grogan et al., 2009) and procedural memory (Ullman, 2004), while the putamen has been implicated in articulatory planning and detecting phonological errors (e.g., Abutalebi and Green, 2007).

While the emphasis on phonological skills in previous studies is understandable, in light of the DP model, it is surprising that the roles played by semantic and verbal episodic memory have seldom been tested in neural research on FLL. Since the DP model was initially proposed, functional imaging and neurological studies have established that semantic functions are supported by a widespread network that includes the ventrolateral prefrontal cortex, anterior temporal lobe, middle temporal gyrus, etc. (Patterson et al., 2007; Binder et al., 2009; Ralph et al., 2017), along with sub-cortical regions like the thalamus. In addition to being a main relay station of sensory information, the thalamus may also play an important role in language production by selecting lexical and semantic representations (Abutalebi and Green, 2016), by virtue of its connection with the left IFG (Ford et al., 2013). The episodic memory is subserved by the hippocampus and its surrounding medial temporal lobe structures (including the entorhinal cortex and the parahippocampal gyrus), along with the prefrontal cortex (e.g., Breitenstein et al., 2005). The hippocampus is well-known for its role in transforming short-term memory to long-term memory, while the entorhinal cortex and the parahippocampal gyrus are implicated during the processing of object/event information and spatial-temporal context, respectively (Eichenbaum et al., 2012). Another study also reported that the entorhinal cortex reflects elementary memory processes related to novelty detection, while the parahippocampus is more involved in the formation and subsequent reactivation of the memory (Daselaar et al., 2004). A few recent studies have reported evidence that supports a role of hippocampus in younger adults in FLL (Kepinska et al., 2017, 2018). For example, the connectivity of the hippocampus and Broca's area was implicated during the acquisition of a novel grammar (Kepinska et al., 2018).

There are reasons that the brain networks and cognitive mechanisms could be different in older learners, which could result in different learning outcomes (Service and Craik, 1993; Glass et al., 2012; Ingvalson et al., 2017). To start with, older adults had poorer speech discrimination for phonemes and pitch (Shen et al., 2016), which may promote the use of alternative strategies in vocabulary learning. Due to lower working memory capacity (Service and Craik, 1993; Mackey and Sachs, 2012), they may also rely less on rote memorization. In addition, the inhibition function is generally in decline (Hasher et al., 1991, but see Veríssimo et al., 2021), making it difficult for them to retrieve the L2 word because it is more difficult for them to suppress the L1 word. In contrast, older adults often have better crystallized knowledge (e.g., world and vocabulary knowledge) than younger adults (Salthouse, 2012; Hartshorne and Germine, 2015). It is plausible that declarative memory may play a crucial role in older learners (Ullman, 2001b). For example, they may take advantage of their better semantic knowledge to remember the vocabularies as a chunk (e.g., by forming a story to connect the words being learnt), or to proactively derive semantic associations for use as retrieval cues in episodic memory recall. However, individual differences studies on FLL in older adults have remained scarce, and even fewer have examined the neural correlates of FLL in older adults. To our knowledge, one such study has focused on vocabulary learning, and it suggested that the hippocampal volume and the associative memory ability prior to language learning are robust predictors of vocabulary proficiency at the end of training (Nilsson et al., 2021). Another study showed that the artificial grammar learning performance was influenced by structural and functional connectivity of the Broca's area and its right hemisphere homolog (Antonenko et al., 2012).

### 1.3. The Present Study

The present study investigates the neurocognitive factors that influence the immediate learning success and long-term retention of the vocabularies during an intensive FLL programme, based on a group of Cantonese-speaking Hong Kong older learners aged 58–69, all of whom were familiar with English as a second language. Italian was chosen as the target language due to its highly regular grapheme–phoneme correspondence, its use of the Latin alphabet, the unfamiliarity with the language by the general population in Hong Kong, and the popularity of Italy as a tourist destination. By design, while both vocabulary and basic grammar rules were taught throughout the learning programme, a stronger emphasis was put on the vocabulary since it plays a fundamental role in both spoken and written language comprehension across all stages of learning (Yum et al., 2014). Also, older learners may be less motivated in learning a new language in its entirety, and they may only wish to learn the essential vocabularies for simple communications and travels (Antoniou et al., 2013; Pfenninger and Polz, 2018). Our main hypothesis is that, for foreign vocabulary learning in older adults, both semantic and episodic memory are more strongly associated with the learning success than phonological skills. To examine this hypothesis, two groups of analyses were conducted, using MRI and cognitive data to predict the immediate learning success and long-term retention.

The first group of analyses (MRI analysis) comprised a *cortical analysis* and a complementary *sub-cortical analysis*. For the cortical analysis, using SBM, four cortical morphological measures (cortical thickness, cortical surface area, cortical volume, and mean curvature) were extracted over a broad range of regions of interest (ROIs); these measures were then used as predictors to test the associations of each ROI with the in-class performances and the final test scores. The ROIs were selected because they have consistently been found to support semantic memory (Patterson et al., 2007; Lau et al., 2008; Binder et al., 2009; Ralph et al., 2017), have exhibited learning-induced neuroplasticity (Lee et al., 2007; Stein et al., 2012; Li et al., 2014), have previously been associated with second language learning performance in general (Ullman, 2016; Tagarelli et al., 2019), or are part of the language control network (Abutalebi and Green, 2007). Although language processing is strongly left-lateralized in the brain, the right hemisphere is also heavily engaged for learning a second language (Hosoda et al., 2013; Bubbico et al., 2019; Qi et al., 2019; Chen et al., 2021). Thus, the homologous areas in both hemispheres were included as ROIs. Our hypothesis would be supported if the cortical measures of the brain regions centrally involved in semantic functions (pars orbitalis and temporal pole) or episodic memory (caudal middle frontal cortex and entorhinal cortex) are more associated with learning performance than those that underlie phonological functions (precentral gyrus and SMG).

For the *sub-cortical analysis*, sub-cortical volume measures were used to predict in-class and final test scores. Due to our relatively small sample size and the already extended set of cortical regions examined, exploration was limited to four ROIs: hippocampus, thalamus, caudate nucleus, and putamen. This analysis complemented the cortical analysis in providing further evidence consistent with the main hypothesis. For example, if the hippocampal volume was associated with learning performance, it would strengthen the view that the episodic memory is associated with FLL in older adults.

In the second group of analyses (*cognitive analysis*), a broad range of cognitive and phonological measures, derived from a cognitive and phonological test battery, were used as the predictors of language learning performance. Behavioral support for our main hypothesis would be obtained if the semantic function score (semantic fluency and picture naming) and verbal episodic memory score (Hong Kong List Learning test) are more important predictors of the learning outcomes than phonological function scores (phonological discrimination and awareness).

## 2. Materials and Methods

### 2.1. Participants

A group of 25 older learners aged 58–69 were initially enrolled into the Italian learning programme. They were native Cantonese speakers, had no known neurological disorders and normal/correct-to-normal vision. All of them had limited exposure to Italian, had visited Italy for no more than a month, and had at least 6 years of prior experience in learning English. The cohort was recruited *via* the Institute of Active Aging, Hong Kong Polytechnic University. Hong Kong version of Montreal Cognitive Assessment (Wong et al., 2009) was used to confirm that they were cognitively normal (mean = 27.9, *SD* = 1.7). The attendance for the intensive Italian learning programme was high, with 21 of 25 participants completing all the 21 possible visits. There were four drop-outs midway through the programme, including three who completed 5 visits and one who completed 10 visits. One female participant was left-handed (handedness = −60) according to the Edinburgh inventory (Oldfield, 1971); only the remaining 20 participants were included in the present analysis. Their mean age, education, and HK-MoCA were 63.7 (SD = 2.9, range = 58.6–69.3), 15.2 (SD = 3.1, range = 11–21), and 27.8 (SD = 1.7, range = 25–30), respectively. All procedures were approved by the Ethical Review Committee, Hong Kong Polytechnic University. Written signed informed consent was obtained from all participants. They were reimbursed HKD 800 for their participation.

### 2.2. Study Description

**Figure 1:**
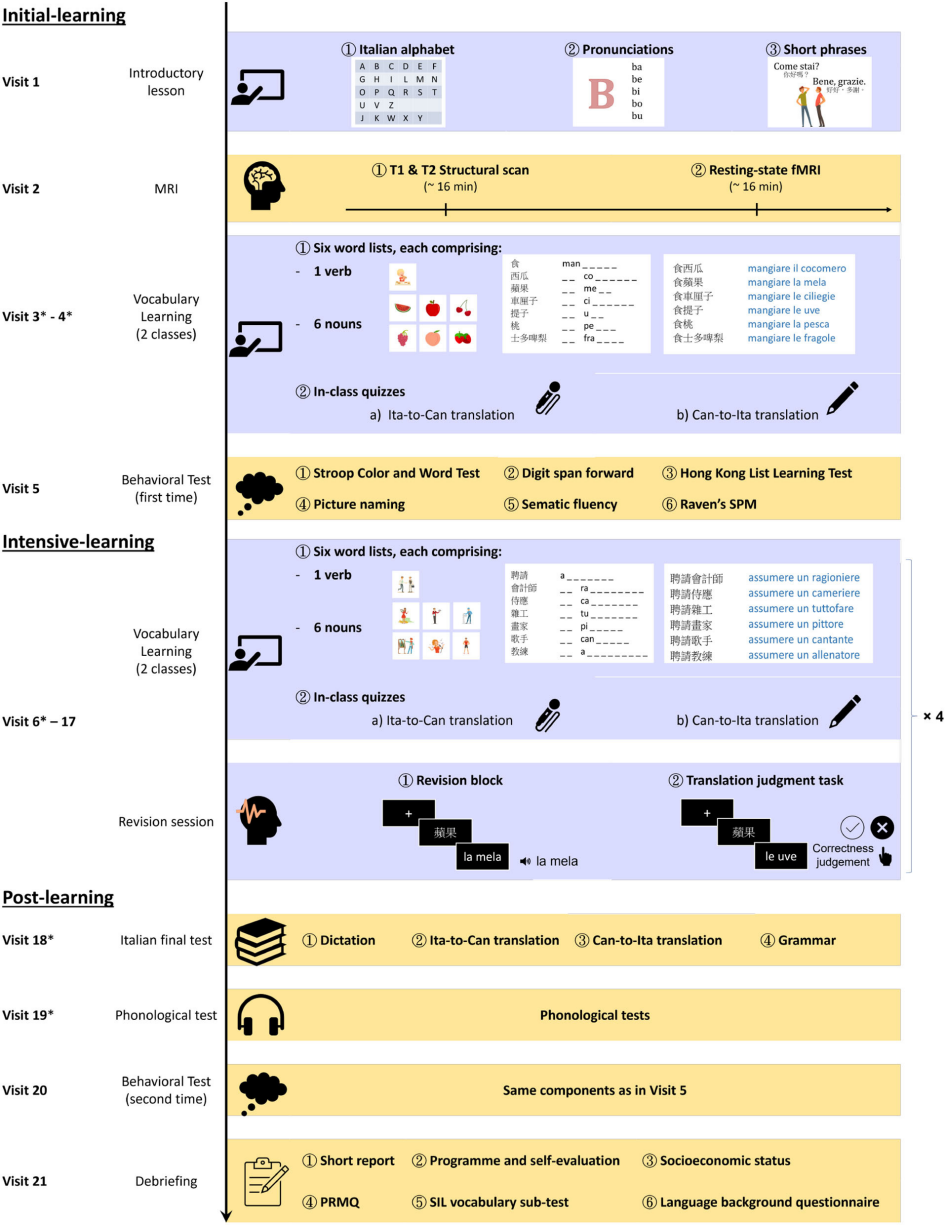
Italian learning programme. The whole programme was divided into three phases—initial-learning (visits 1–5), intensive-learning (visits 6–17), and post-learning (visits 18–21). In the initial- and intensive-learning phases, six word lists were taught per vocabulary lesson, each comprising a verb and six nouns. After two vocabulary lessons, there was a revision session. Such a cycle of three sessions was repeated four times during the learning programme (hence × 4 in the figure). Asterisks (*) were used to indicate the visits during which phonological tests were administered (see main text).

The participants individually attended an intensive Italian vocabulary learning programme (Figure 1). The whole programme comprised an introductory lesson (visit 1), ten computerized vocabulary lessons (visits 3–4, 6–7, 9–10, 12–13, and 15–16), four revisions sessions (visit 8, 11, 14, 17), an Italian final test (visit 18), and a range of tests arranged at various points of the programme. These included an MRI session (visit 2), initial-learning phase cognitive tests (visit 5), initial-learning phase phonological tests (at the beginning of visits 3, 4, and 6), post-learning phase phonological tests (visits 18–19), post-learning phase cognitive tests (visit 20), and a debriefing session (visit 21; see Supplementary Section S1). The MRI, phonological, and cognitive testing were not conducted right at the beginning, for two reasons. (1) In reality, many language learners have some degree of knowledge of the target foreign language before proceeding to take a formal course. The introductory session serves to provide a more uniform experience prior to the MRI visit. Also, the introductory session gave them a taste of learning a new foreign language and offered a buffer period for them to commit to participating in the structural MRI session and the subsequent intensive Italian learning programme, which would likely reduce the dropout rate. (2) Upon enrolling to the study, most participants were anxious and most motivated to start learning Italian. As such, arranging too many tests that are unrelated to Italian learning right at the beginning would likely be seen as non-ideal arrangement from the learner's view, which might increase the dropout rate. Hence, the phonological and cognitive testing were not arranged immediately after the MRI session, but they were spread over sessions 3–5 as a compromise, especially because it was not our primary goals to investigate learning-induced cognitive gains. Overall, the whole programme can be divided into three phases—initial learning (visits 1–5), intensive-learning (visits 6–17), and post-learning (visits 18–21). The division of visits 1–17 into two phases was an arbitrary one, but the main difference was that a variety of tests were throughout the initial learning stage, while a revision session was arranged every two lessons only during the intensive-learning phases. Also, there was more separation between visits in the initial-learning phase (4.5 days on average, *SD* = 1.1 days) than in intensive-learning (3.2 days, *SD* = 0.7 days), due to scheduling difficulties. The completion time of the first twenty visits of the programme was 68.9 days on average (*SD* = 11.6 days). The details for each component are laid out below in the following sections: Italian vocabulary learning programme, MRI protocol, and behavioral test battery.

### 2.3. Italian Vocabulary Learning Programme

#### 2.3.1. Introductory Lesson

Each participant attended a 1.5-h introductory Italian lesson, in which they watched a custom-made video under the direction of an experimenter. There were three main parts in the video: Italian alphabet, Italian sounds, and Italian short phrases. Sixteen syllable identification questions were given as homework.

#### 2.3.2. Computerized Vocabulary Lessons

Each computerized vocabulary learning lesson was about 120 min long. The goal of each lesson was to learn six word lists, each comprising a transitive verb (e.g., *mangiare*, “to eat”) and six matching nouns (e.g., *la mela*, “an apple.”) Thus, over the course of ten vocabulary lessons, there were 60 word lists in total, comprising 60 verbs and 360 nouns (see Supplementary Table 1, for the complete list of Italian words used, along with their Cantonese and English translation). In constructing these word lists, control measures were taken so that any systematic changes in the learning performance over time would not be due to confounding factors like the inherent difficulties of the Italian word lists or their Cantonese counterparts. In particular, because Italian words with a larger number of syllables or letters would likely be more difficult to remember, 5 groups of 12 word lists were constructed, with each group being the study materials for two consecutive classes. Across all pairs of groups, the verbs and nouns were matched separately in number of syllables (verbs: *p*s >0.14, nouns: *p*s >0.10) and letters (verbs: *p*s >0.32; nouns: *p*s >0.14). The Cantonese translation of the word list were also matched in the number of Chinese characters (verbs: *p*s >0.10; nouns: *p*s >0.07) and strokes (verbs: *p*s >0.28; nouns: *p*s >0.12). The stimulus matching was not conducted by lesson, because, due to the non-arbitrary pairing of the verbs and nouns, it was exceedingly difficult to create 10 groups that are matched in all four variables. Dividing by week also provided a better match in terms of the structure of the whole programme, in that, during the intensive-learning phase, the vast majority of older adults had two lessons per week, and a revision session was arranged every two lessons. By using week rather than lesson as a measurement unit, each in-class score is the average over two lessons, which should have lower measurement noise compared to that derived using only one lesson. For more details about the stimulus matching and construction, see Supplementary Section S1 and Supplementary Table S1. The teaching materials were delivered based on a custom-made E-Prime 3.0 script.

For each word list, the participant first learnt the verb through a picture depicting the verb and by listening to its pronunciation three times. Next, the participant learnt the six nouns through clicking on the corresponding pictures to listen to their pronunciations. Each picture could be clicked three times, before it would vanish on-screen. The order and pace of learning were self-determined. The participant was then given a chance to read aloud the seven words, upon hearing the pronunciation of each word. Next, they completed a fill-in-the-blank exercise on paper, in which they should spell all the seven words learnt. They were asked to check their own answer directly afterwards. The participant then listened to the pronunciation of the six verb-noun phrases (e.g., *mangiare la mela*, meaning “to eat an apple”) for three times each, before continuing with the next word list. The learning of each list usually took 15–20 min. After learning all six word lists, a short quiz was given at the end of each lesson. They were given as much time as they wish before the short quiz. The fill-in-the-blank and the quiz were included to promote memory consolidation and to assess immediate learning success, respectively.

There were two parts in the quiz: Italian-to-Cantonese and Cantonese-to-Italian translation (*abbr*. Ita-to-Can and Can-to-Ita thereafter). In either part, all 36 phrases learnt were tested, with each verb and noun would be tested six times or once, respectively. In Ita-to-Can, the participants read a verb-noun phrase (e.g., *mangiare la mela*) on screen and listened to its pronunciation, and they were asked to orally translate the phrase into Cantonese. The maximum score for this part was 36 × 2 = 72. In Can-to-Ita, the participants were given a paper-and-pen test, and they should write down the Italian translation of Cantonese verb-noun phrases. The order of the phrases was pseudorandomized, with no more than two consecutive phrases sharing the same verb. No feedback about the correct answer was given. For simplicity, participants were asked to write down the taught form of article (definite or indefinite) for the nouns in Can-to-Ita. However, the article was not scored because there was more than one possible grammatically correct choices, and their distinction was only learnt gradually; thus the maximum score remained as 72. As such, the grammatical use of the article was only scored in the final test.

In addition, six pages of handouts (one word list per page) showing the concepts learnt in pictorial form were given at the end of each lesson, on which the participants should copy the Italian words, either immediately or at home. Though the lexical consolidation process would be influenced by the number of times that participants revised the Italian vocabularies, the notes were essential for the effectiveness of the lessons. Also, although these lessons put more emphasis on vocabulary building, basic grammatical components and rules (including gender, singular and plural forms, article, present tense, adjective, numerals, and possessive adjective) were also taught, through four handouts and corresponding homework exercises, distributed at the end of lesson 1, 6, 8, and 10. Participants attended four revision sessions in total, each time following the completion of two vocabulary sessions. Results for the revision sessions, which involved electroencephalogram recordings, will be reported in a separate paper (under preparation).

#### 2.3.3. Italian Final Test

An 1.5-h Italian test comprising four different parts—dictation, Ita-to-Can, Can-to-Ita, and grammar—was administered about one week after the final vocabulary lesson. In the first three parts, the words were uniformly sampled across the ten vocabulary learning lessons. The first three parts were delivered using E-Prime 3.0, while the grammar test was an open-note, paper-and-pen test. For dictation, 60 nouns (including the article, e.g., il gesso, “the chalk”) were presented aurally at a rate of 15 s / item. Participants were asked to dictate each word on an answer sheet. The nouns would be repeated at a rate of 5 s / item, during which they could check and change their answers. Each correct spelling of the article and noun was worth half a point. The procedures for Ita-to-Can and Can-to-Ita translation were the same as those for the vocabulary lessons, except that there were only 30 verb-noun phrases here. While there were 30 questions in each test, the maximum score of Ita-to-Can was 30 × 2 = 60 because participants were asked to translate one Italian verb-noun phrase into one Cantonese verb and one Cantonese noun; the Cantonese verb and noun accounted for a point each. The maximum score of Can-to-Ita was 30 × 3 = 90 because participants were asked to translate one Cantonese verb-noun phrase into a verb, an article, plus a noun. For the grammar test, the questions were multiple-choice questions with either two or four choices; correspondingly, each question was worth two or four marks. The distribution of points approximately matched the relative emphasis in the grammatical notes. For the details of the final test, see Supplementary Section S1 and Supplementary Table S2.

#### 2.3.4. Measures of Immediate Learning Success and Language Retention

The participants' performance in each lesson was summarized by means of two scores: Ita-to-Can translation (maximum: 42) and Can-to-Ita translation (maximum: 42). For the final test, the maximum for dictation, Ita-to-Can, Can-to-Ita, and grammar were 60, 60, 90, and 180, respectively. Partial credit was not given; the Italian spellings or Cantonese words should be produced entirely.

### 2.4. MRI Protocol

#### 2.4.1. Structural MRI

The MRI session was arranged after the introductory lesson. All patients were scanned with a Signa Premier 3T scanner (GE Healthcare, USA), located at the MRI Unit, Department of Radiology, University of Hong Kong. A head coil with forty-eight channels was used. High-resolution T1-weighted structural scans were acquired using a T1-weighted sequence known as 3D Gradient-Echo BRAin VOlume (BRAVO; TR 7.3 ms, TE 3 ms, TI 900 ms, flip angle 8°, 1 × 1 × 1 mm^3^ voxels, TA = 5 min 42 s, FOV = 256 × 256 × 376 mm^3^). T2 and resting-state fMRI data were also acquired, but they were not analyzed for the present paper. The total acquisition time was about 35 min.

#### 2.4.2. Extraction of Anatomical Measures

The T1 data were analyzed using FreeSurfer 7.2.0 recon-all segmentation pipeline (Fischl, 2012). The automatic pipeline started with various pre-processing steps, including motion correction, skull-stripping, removal of non-brain tissues, and intensity normalization. The pipeline then proceeded with both surface-based morphometry (SBM) and volume-based morphometry.

**Figure 2:**
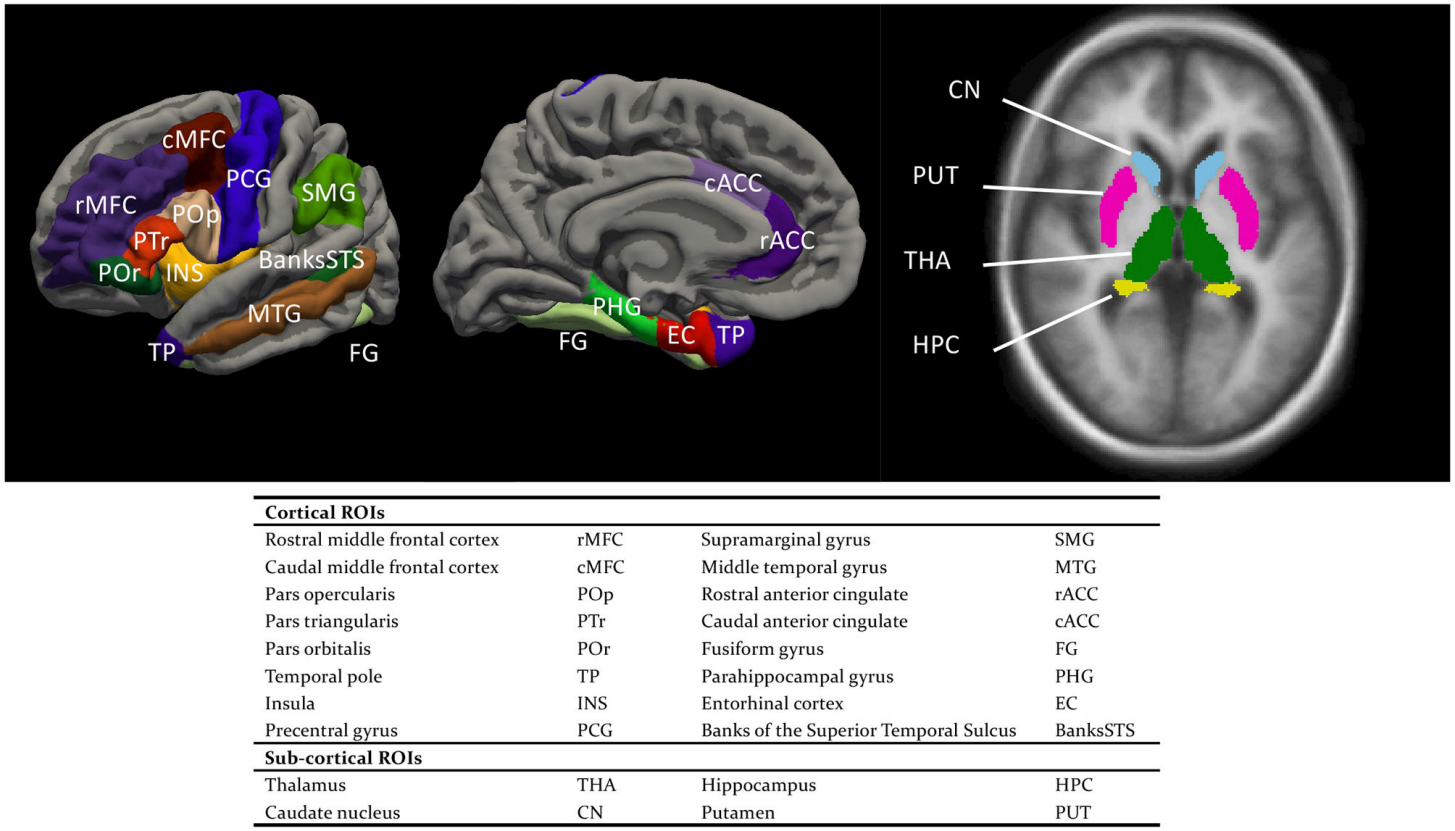
Cortical and sub-cortical regions of interest (ROIs).

Regarding the SBM part, a mesh model was constructed for the cortical surface, comprising an inner white matter surface that separated gray matter (GM) and white matter (WM) and an outer pial surface that separated GM and cerebrospinal fluid (Fischl and Dale, 2000). The surface models were then inflated, and co-registered to the fsaverage template. Desikan-Killiany cortical parcellation (aparc.annot) was adopted for cortical segmentation (Desikan et al., 2006). For each cortical ROI (Figure 2), four measures were extracted based on the surface models by summing/averaging across the vertices of the mesh: cortical thickness (the mean distance between the white and pial surface), cortical surface area (the sum of areas, measured at the white surface), cortical volume (the total volume of the gray matter that lies between the white and pial surface), and mean curvature (the average degree of cortical gyrification) (Winkler et al., 2010; Luders et al., 2012). Our rationale for using all four cortical measures was three-fold. (1) They have all been implicated in either language learning or cognition in general. (2) As a group, they would multivariately explain a larger percent of variance in the learning performance than they would individually, given that they likely encapsulate complementary information (e.g., Winkler et al., 2010; Yang et al., 2016). For example, there is only a weak correlation between cortical thickness and cortical surface area (Winkler et al., 2010). (3) These measures also have different developmental trajectories across the lifespan (Hogstrom et al., 2013), differential associations with cognitive functions (Gautam et al., 2015; Chung et al., 2017; Green et al., 2018; Tadayon et al., 2020) or even genetic factors (Grasby et al., 2020). For example, not only were individual differences in cortical thickness and cortical surface area ascribed to largely different genetic factors, but these factors also exert their influences in different developmental stages (Grasby et al., 2020).

The volume-based morphometry involves a non-linear volumetric registration to the FreeSurfer atlas, with the structural labeling being performed using a Gaussian Classifier Atlas (GCA; Fischl et al., 2002). For each sub-cortical ROI (Figure 2), the sub-cortical volume was extracted as the single measure.

### 2.5. Behavioral Test Battery

#### 2.5.1. Cognitive Test Battery

A neuropsychological test battery comprising six standard cognitive tests was conducted, with the language of instruction being the native language of the participants (Cantonese). These tests included the Stroop Color and Word Test (Golden and Freshwater, 1978; Fong et al., 2020), digit span forward, Hong Kong List Learning Test (HKLLT; Chan and Kwok, 1998), picture naming (Bates et al., 2003; Fong et al., 2020), semantic fluency (Fong et al., 2021), and Raven's Standard Progressive Matrices (Raven and Court, 1998), and they were administered after three Italian learning lessons and one week post-learning. These tests assess processing speed, inhibition, short-term memory, verbal episodic memory, semantic knowledge, and matrix reasoning, respectively (Table 1). This battery was administered at the fifth visit (initial-learning phase) and the twentieth visit (post-learning phase) to assess the learning-induced cognitive gains, except for the possible use of two different versions of tests to minimize training effects. Details of this battery can be found in Supplementary Section S2 and Supplementary Tables S3–S5.

**Table 1:** Measures of behavioral test performance.

**Task**	**Cognitive function or phonological skill**	**Detail**
Stroop Color and Word Test	Processing speed	Average number of correct responses in 45 s across the word and color sub-tasks
	Inhibition function	Number of correct responses in the color-word sub task, divided by the processing speed measure above
Digit span forward	Phonological STM	The maximum memory load reached (test continued if 2 of 3 questions were correct at a given load; half a point would be added in case of a single correct answer)
Hong Kong List Learning Test	Verbal episodic memory	Number of memory items retrieved after 30 min
Picture naming task	Naming latency	The average latency of correct answers
Semantic fluency	Semantic memory	Average number of responses across 16 semantic categories
Raven's SPM	Matrix reasoning	Standardized number of correct responses
Cantonese discrimination	Cantonese perceptual skill	Number of correct answers (Max: 41)
Cantonese Spoonerism	Cantonese phonological awareness	Number of correct answers (Max: 30)
English discrimination	English perceptual skill	Number of correct answers (Max: 38)
English Spoonerism	English phonological awareness	Number of correct answers (Max: 30)
Italian discrimination	Italian perceptual skill	Number of correct answers (Max: 35)
Italian Spoonerism	Italian phonological awareness	Number of correct answers (Max: 30)

#### 2.5.2. Phonological Test Battery

Two phonological tests (discrimination and Spoonerism) each were run for all three languages—Cantonese (L1), English (L2), and Italian (L3). For the discrimination task, participants heard four auditory stimuli in succession in each trial (*c.f*. Koda, 1998). Three of the stimuli shared the same segmental or suprasegmental feature (in the case of Cantonese tone), with the fourth not sharing the feature in question. The number of blocks and trials for each language examined was different, due to the different number of distinctive segmental and suprasegmental feature in each language. The Cantonese discrimination task was divided into three blocks, in which the feature in question was initial consonant (25 trials), vowel (10 trials), and tone (6 trials), respectively. The English discrimination task was divided into two blocks, in which the feature in question was initial consonant (26 trials) and vowel (12 trials). The Italian discrimination task was divided into two blocks, in which the feature in question was also initial consonant (25 trials) and vowel (10 trials). For Spoonerism, participants listened to a pair of words in succession. Their task was to swap the initial consonant of the two words. There were 15 trials in all three versions. The test would be terminated if the participants failed to score any point in three consecutive trials. The Cantonese and English tests were arranged on visits 3 and 4 (one per visit, order counterbalanced), and together in visit 19 (order counterbalanced). The Italian tests were arranged on visits 5 and 18. More details of this battery can be found in Supplementary Section S3 and Supplementary Tables S6–S9.

### 2.6. Linear Mixed-Effects Modeling

Linear mixed-effects (LME) models were constructed to test our main hypothesis that semantic and episodic memory functions play a major role in language learning in older learners. First, baseline demographic models (baseline models thereafter) were constructed for each of the three scores (in-class Ita-to-Can, in-class Can-to-Ita, and final test). In each baseline model, in addition to either Week (for the in-class Ita-to-Can or Can-to-Ita) or TestPart (for the final test), three demographic variables (age, years of education, and gender) were included as fixed-effects predictors, while participants were included as a random-effects variable.

Next, two groups of analyses were conducted, each involving comparisons between the full model(s) of interest against the corresponding baseline models. The first analysis comprised both a cortical and a complementary sub-cortical analysis. In the cortical analysis, the surface-based morphological measures were used as predictors to test the neural associations with the in-class performances and the final test scores. The regions of interest (ROIs), illustrated in Figure 2, comprised the Broca's area (pars opercularis and pars triangularis), the regions supporting semantic memory (pars orbitalis, temporal pole, and middle temporal gyrus) and episodic memory (caudal and rostral middle frontal cortex, entorhinal cortex, and parahippocampal gyrus), as well as regions important for phonological processing (SMG, precentral gyrus, banks of superior temporal sulcus), procedural memory (insula), language switching (caudal and rostral anterior cingulate), and orthographical processing (fusiform gyrus). For each ROI, a full model was constructed that include all the predictors in the baseline model, four anatomical measures of the ROI, and the interactions between each anatomical measure with either Week or TestPart. Likelihood ratio tests were used to evaluate whether the full model of each ROI represented an improvement over the baseline model. Multiple comparisons were corrected using the false discovery rate (FDR) procedure. If a significant interaction was found, *post hoc* trend analysis was conducted to reveal how the degree of association of each anatomical measure with the language learning performance was modulated by Week or TestPart. In the sub-cortical analysis, the sub-cortical volumes were used to predict the in-class and final test scores. The eight regions of interest (ROIs) included the hippocampus, thalamus, caudate nucleus, and putamen on both hemispheres. For each ROI, a full model was constructed using the sub-cortical volume of the ROI and its interaction with either Week or TestPart. The subsequent likelihood ratio tests were FDR-corrected.

In the second group of analyses—cognitive analysis—the cognitive predictors, derived from the neuropsychological data, were included as the additional predictors for modeling the three scores (Bonferroni correction was applied). For each model, backward elimination was conducted to yield the final model.

### 2.7. Cognitive and Phonological Skill Comparisons Between Initial- and Post-learning

For completeness, we tested the changes in cognitive functions and phonological skills incurred between the initial- and post-learning, despite the lack of a control group to assess whether the changes were due to repetition effects or induced by language learning. For each cognitive/phonological measure, a pairwise Wilcoxon signed rank test (two-tailed) was conducted with FDR-correction.

## 3. Results

### 3.1. Italian Learning Performances

**Figure 3:**
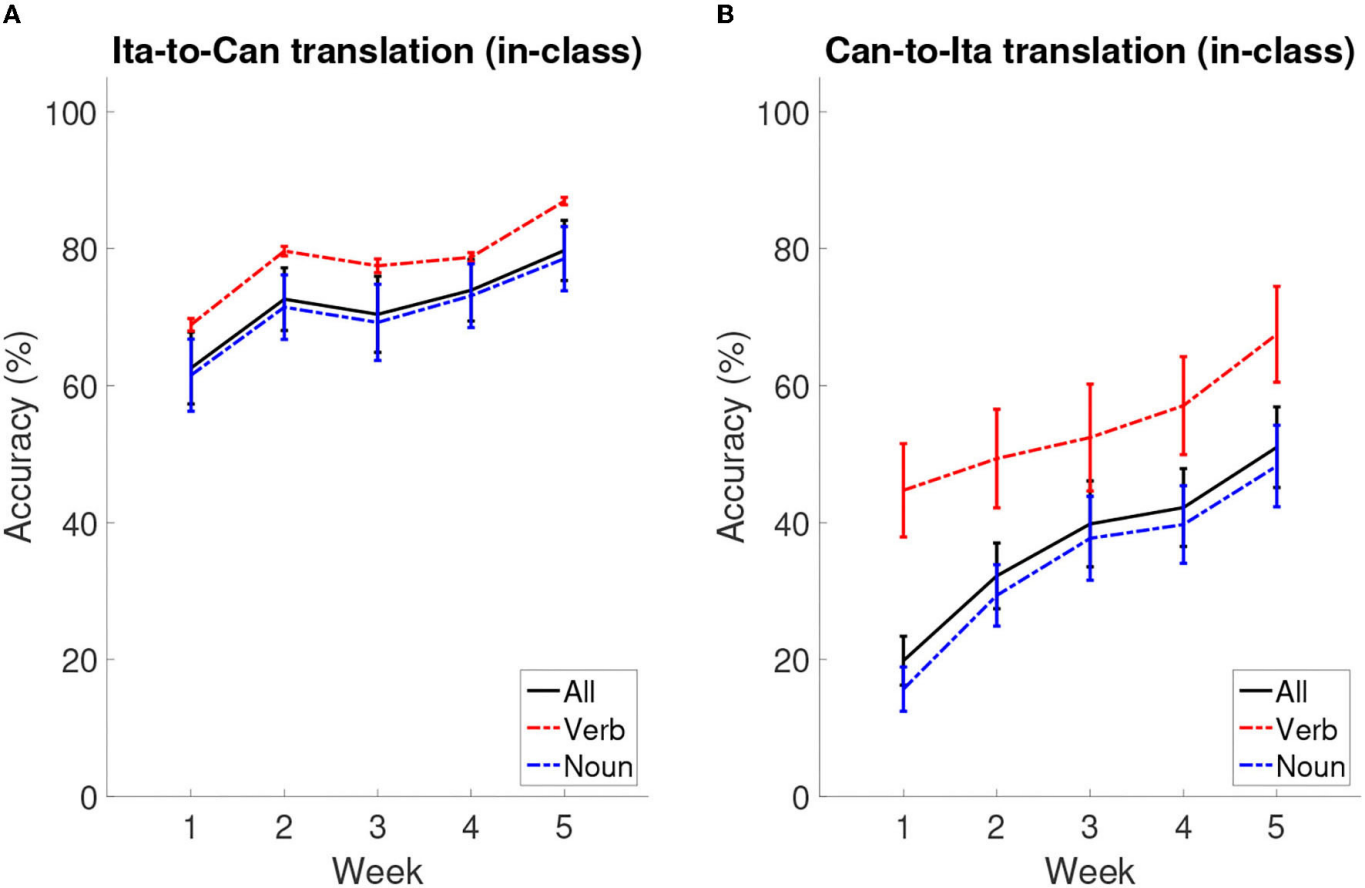
Learning curves across the five weeks. **(A)** The accuracy of Ita-to-Can translation across weeks. **(B)** The accuracy of Can-to-Ita translation across weeks. Solid lines: overall in-class learning performance as a function of week; dotted lines: the scores for the verb and noun components, separately plotted to reflect the structure of the in-class quizzes. The error bars represent standard error means.

Table 2 shows the summary statistics for the mean performance for the two in-class quizzes (Ita-to-Can and Can-to-Ita) and the final test, which were used as indices for immediate learning success and language retention, respectively. Figure 3 illustrates the overall in-class learning performance across the five weeks; the verb and noun components were separately plotted to reflect the structure of the in-class quizzes, although only the overall learning performance was selected for statistical analysis. To test whether the improvement was significant across the five weeks, a linear mixed-effects model was fitted for each in-class quiz, using the demographic variables for predicting the performance (Table 3). In both cases, the main effect of Week was significant, *p*s < 0.001. For Ita-to-Can, *post hoc* pairwise comparisons with Tukey's correction showed that the performance for week 1 was significantly worse than all other weeks, while that for week 5 was significantly better than all other weeks, with no differences among Week 2–4 being significant. For Can-to-Ita, the *post hoc* pairwise comparisons indicated that the improvements over two weeks or more were all significant, although the improvement between successive weeks was only significant going from Week 1 to 2. These results suggested that the participants were generally able to learn an increasing number of Italian words across the five weeks.

**Table 2:** Summary statistics of the scores for the in-class quizzes (Ita-to-Can and Can-to-Ita) and the final test.

**Score (%)**	***Mean***	***SD***	***Min***	***Max***
InClass-Ita-to-Can	71.8	20.4	26.7	93.5
InClass-Can-to-Ita	41.1	22.5	6.2	73.0
FinalTest-Dictation	59.5	17.1	30.0	91.7
FinalTest-Ita-to-Can	80.2	21.4	36.7	100
FinalTest-Can-to-Ita	56.4	27.7	16.7	96.7
FinalTest-Grammar	92.9	5.9	78.9	100

**Table 3:** Demographic models for the in-class quizzes (Ita-to-Can and Can-to-Ita) and the final test.

**Score**	**Term**	**SumSq**	**MeanSq**	**NumDF**	**DenDF**	**F**	**p**	
Ita-to-Can	Week	3156.37	789.09	4	80.0	19.83	<0.001	***
	Age	29.23	29.23	1	20.0	0.73	0.402	ns
	Education	25.39	25.39	1	20.0	0.64	0.434	ns
	Gender	21.36	21.36	1	20.0	0.54	0.472	ns
Can-to-Ita	Week	4726.65	1181.66	4	80.0	20.64	<0.001	***
	Age	68.48	68.48	1	20.0	1.20	0.287	ns
	Education	43.20	43.20	1	20.0	0.75	0.395	ns
	Gender	0.59	0.59	1	20.0	0.01	0.920	ns
FinalTest	TestPart	18082.02	6027.34	3	60.0	41.41	<0.001	***
	Age	307.16	307.16	1	20.0	2.11	0.162	ns
	Education	119.81	119.81	1	20.0	0.82	0.375	ns
	Gender	132.84	132.84	1	20.0	0.91	0.351	ns

*** *, p < 0.001*,

** *, p < 0.01*,

* *, p < 0.05*,

† *, marginal, ns, non-significant*.

For the final test, the participants were able to dictate 59.5% of the Italian words, backward translate 80.2% of Italian words into Cantonese verbally, forward translate 56.4% of Cantonese words into Italian in written form, and achieve a score of 92.9% for grammar. It is worth noting that the first three parts were closed-note tests while participants were allowed to refer to the notes in the grammar part, which likely accounts for the higher grammar score. In the baseline model, the main effect of TestPart was significant, *p* < 0.001. *Post hoc* pairwise comparisons showed that the test score significantly differed across the four parts, except between dictation and Can-to-Ita.

### 3.2. MRI Results

#### 3.2.1. Surface-Based Cortical Measures and Brain Volume Measures

The four cortical anatomical measures (thickness, surface area, volume, and mean curvature) and the sub-cortical volumes were extracted for all cortical and sub-cortical ROIs (see Supplementary Section S4 and Supplementary Tables S10, S11). In general, the cortical measures of the same ROI were strongly correlated, suggesting a high degree of redundancy in the measures. Despite the expected shared variance among the predictors, the associations with the learning performances could be best captured multivariately by the whole set of parameters. Therefore, all four predictors were included in testing the associations of the ROI with Italian learning performances. Special considerations were taken in interpreting the coefficients of the predictors (see section 3.2.2).

#### 3.2.2. Cortical Predictors of Learning Performance

LME modeling was conducted on the in-class and final test scores. Table 4 summarizes the cortical ROIs at which the anatomical parameters were sensitive to the learning performance (for the results at all the ROIs tested, see Supplementary Tables S12, S14, and S16). For transparency, the marginally significant associations (FDR-corrected *p* < 0.10) and all other associations with uncorrected *p* < 0.05 were also tabulated. Due to the strong correlations among the four predictors, sequential regression was applied to transform the original predictors into four adjusted predictors (more precisely, the most important predictor, determined by the chi-square test, was kept unchanged; in other words, only three predictors were transformed). In this way, the same amount of variance was explained by these adjusted predictors and the original predictors, with the advantage of improved interpretability of the predictors. Type III sums of squares procedure was conducted to estimate each fixed-effect term, including the main effects and the four interaction terms (Supplementary Tables S13, S15, and S17). *Posthoc* trend analysis was conducted on the significant interactions using the Satterthwaite method for estimating degrees of freedom. For in-Class Ita-to-Can score, after FDR correction for multiple comparisons, the baseline model was only marginally improved by the anatomical measures of four ROIs: (1) left pars orbitalis, *p* = 0.052; (2) left caudal middle frontal cortex, *p* = 0.075; (3) right insula, *p* = 0.075; and (4) left entorhinal, *p* = 0.082 (see Supplementary Section S5, for the follow-up analyses on each ROI).

**Table 4:** Cortical ROIs showing significant associations with the Italian learning performance.

**Score**	**Region**	**AIC**	**BIC**	**logLik**	***r2m***	***r2c***	**χ^2^**	**df**	**p (unc)**	**p (FDR)**	
Ita-to-Can	L pars orbitalis	742.99	821.14	−341.49	0.67	0.94	43.75	20	0.002	0.052	†
	R insula	747.47	825.63	−343.74	0.75	0.93	39.26	20	0.006	0.075	†
	L caudalmiddlefrontal	747.91	826.07	−343.96	0.63	0.94	38.82	20	0.007	0.075	†
	L entorhinal	749.27	827.42	−344.63	0.58	0.94	37.46	20	0.010	0.082	†
	R parsorbitalis	751.16	829.31	−345.58	0.33	0.94	35.58	20	0.017	0.110	ns
	L parstriangularis	751.90	830.06	−345.95	0.59	0.93	34.83	20	0.021	0.112	ns
	L middletemporal	754.23	832.38	−347.11	0.49	0.94	32.51	20	0.038	0.163	ns
	R rostralmiddlefrontal	754.50	832.65	−347.25	0.53	0.93	32.23	20	0.041	0.163	ns
	R entorhinal	755.31	833.46	-347.65	0.42	0.94	31.43	20	0.050	0.177	ns
Can-to-Ita	L parsorbitalis	739.46	817.62	−339.73	0.60	0.96	79.54	20	<0.001	<0.001	***
	R parsorbitalis	773.79	851.94	−356.89	0.36	0.94	45.22	20	0.001	0.016	*
	R insula	775.95	854.10	−357.97	0.72	0.92	43.05	20	0.002	0.019	*
	R caudalanteriorcingulate	776.55	854.71	−358.28	0.32	0.94	42.45	20	0.002	0.019	*
	L entorhinal	778.46	856.61	−359.23	0.70	0.92	40.55	20	0.004	0.027	*
	L caudalmiddlefrontal	779.93	858.08	−359.96	0.56	0.93	39.08	20	0.007	0.035	*
	R middletemporal	781.39	859.55	−360.70	0.33	0.94	37.61	20	0.010	0.045	*
	L parstriangularis	782.35	860.51	−361.18	0.47	0.93	36.65	20	0.013	0.052	†
	L temporalpole	782.89	861.04	−361.44	0.64	0.92	36.11	20	0.015	0.053	†
	R parstriangularis	784.01	862.16	−362.00	0.30	0.94	34.99	20	0.020	0.064	†
	R entorhinal	785.88	864.03	−362.94	0.49	0.93	33.13	20	0.033	0.089	†
	R bankssts	785.97	864.12	−362.98	0.41	0.93	33.04	20	0.033	0.089	†
Final test	R entorhinal	659.81	719.36	−304.91	0.75	0.86	48.06	16	<0.001	0.001	**
	L parsorbitalis	665.77	725.32	−307.89	0.71	0.86	42.10	16	<0.001	0.006	**
	L bankssts	668.01	727.57	−309.01	0.69	0.86	39.86	16	0.001	0.009	**
	R fusiform	668.87	728.42	−309.43	0.74	0.83	39.01	16	0.001	0.009	**
	L supramarginal	672.66	732.22	−311.33	0.70	0.84	35.21	16	0.004	0.024	*
	L precentral	673.41	732.96	−311.70	0.67	0.85	34.47	16	0.005	0.025	*
	L parstriangularis	674.62	734.17	−312.31	0.67	0.84	33.26	16	0.007	0.031	*
	L rostralmiddlefrontal	676.83	736.38	-313.41	0.68	0.83	31.05	16	0.013	0.048	*
	L caudalmiddlefrontal	676.87	736.43	−313.44	0.70	0.82	31.00	16	0.013	0.048	*
	R supramarginal	677.77	737.32	−313.89	0.65	0.84	30.10	16	0.017	0.056	†

*p(unc), uncorrected p, p(FDR), FDR-corrected p*.

*** *, p < 0.001*,

** *, p < 0.01*,

* *, p < 0.05*,

† *, marginal, ns, non-significant*.

**Figure 4:**
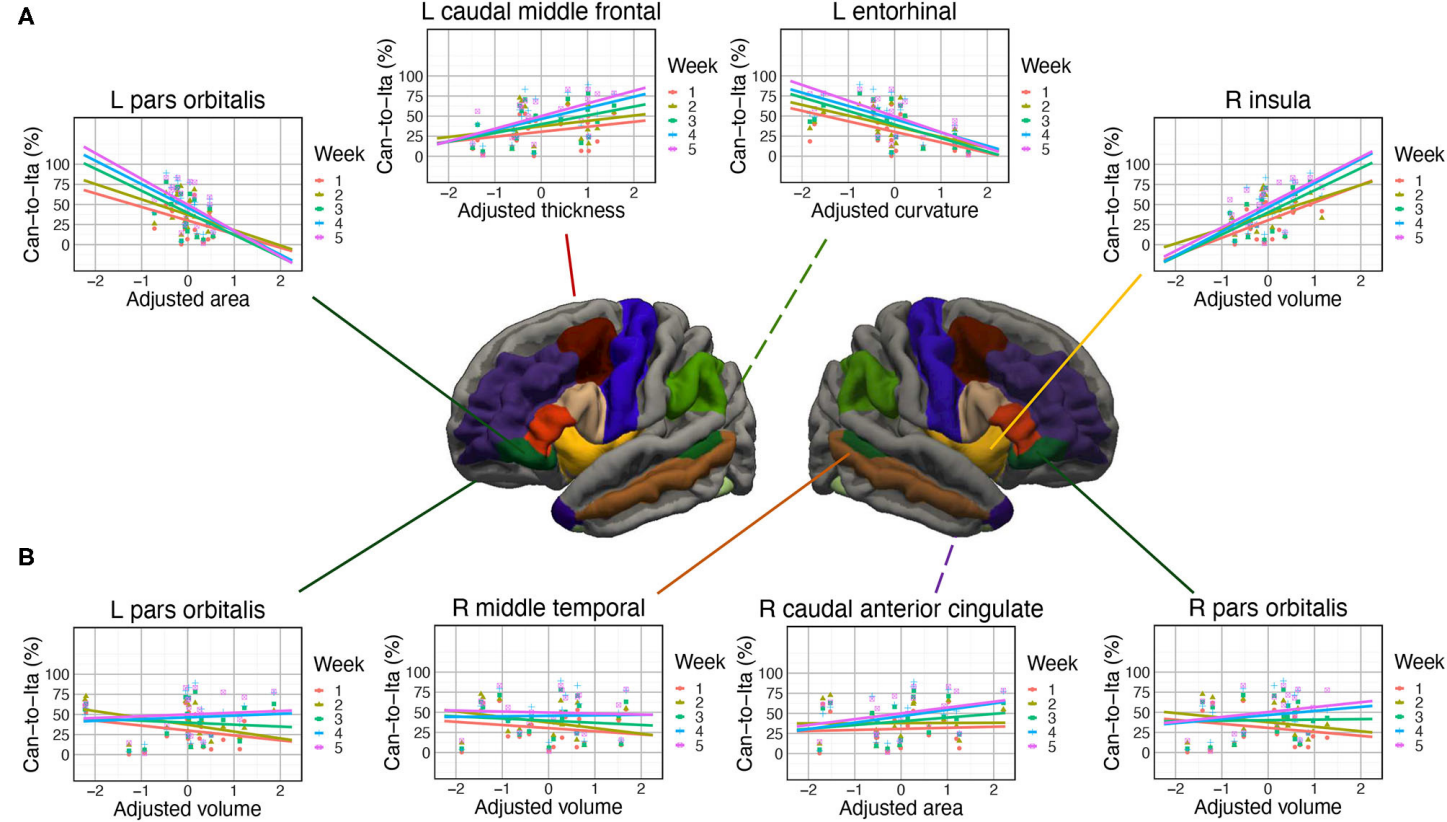
Associations of the in-class Can-to-Ita score with the cortical measures. **(A)** Significant associations at left pars orbitalis, left caudal middle frontal cortex, left entorhinal cortex, and right insula. **(B)** Subtle modulations in the degree of association between final test scores with the cortical measures, found at left pars orbitalis, right middle temporal gyrus, right caudal anterior cingulate, and right pars orbitalis.

The prediction of in-class Can-to-Ita performance was significantly improved by the cortical anatomical measures of seven ROIs. For (1) left pars orbitalis, the performance was significantly associated with adjusted curvature, *t* = 3.03, *p* = 0.007, and adjusted area, *t* = −2.35, *p* = 0.029. Week × adjusted area was significant, with the negative association being significant only for Week 3–5, and that the association was significantly larger in magnitude in Week 5 than both Weeks 1 and 2 (Figure 4A). Week × adjusted volume was also significant. Although the association was non-significant in all weeks, there were subtle pairwise differences in the strength of association across different weeks. For (2) right pars orbitalis, Week × adjusted volume was significant, but the *post hoc* trend analysis revealed no significant association. Like left pars orbitalis, the interaction was due to subtle pairwise differences in the strength of association across different weeks. For conciseness, only significant associations were reported thereafter. For (3) right insula, the performance was significantly predicted by adjusted volume, *t* = 4.10, *p* < 0.001, adjusted area, *t* = −3.95, *p* < 0.001, and adjusted curvature, *t* = −2.58, *p* = 0.018. Week × adjusted volume was significant, with the association being significant in all five weeks; the interaction was due to the significantly larger association strength in the final two weeks (Weeks 4 and 5) than Week 2. For (4) right caudal anterior cingulate cortex, both Week × adjusted area and Week × adjusted curvature were significant, but none of the association was significant in any of the weeks. For (5) left entorhinal cortex, the performance was significantly associated with adjusted thickness, *t* = 3.62, *p* = 0.001, and adjusted curvature, *t* = −5.16, *p* < 0.001. There was a significant Week × adjusted curvature, which was due to the much more negative association in Week 5 than in both Week 1 and 2. For (6) left caudal middle frontal cortex, there was significant association between Can-to-Ita performance with adjusted curvature *t* = 2.75, *p* = 0.013, and with adjusted thickness *t* = 2.48, *p* = 0.022. Week × adjusted thickness was significant, which was attributed to the fact that the association was significant only for Week 3–5. For (7) right middle temporal gyrus, Week significantly interacted with adjusted area and adjusted volume. However, the association between performance and neither measure was significant in any of the weeks. For an understanding about the subtle changes in association across weeks, readers may refer to Figure 4B. Apart from the seven ROIs above, the baseline model was marginally improved at five additional ROIs (left pars triangularis, left temporal pole, right pars triangularis, right entorhinal cortex, and right banks of STS).

**Figure 5:**
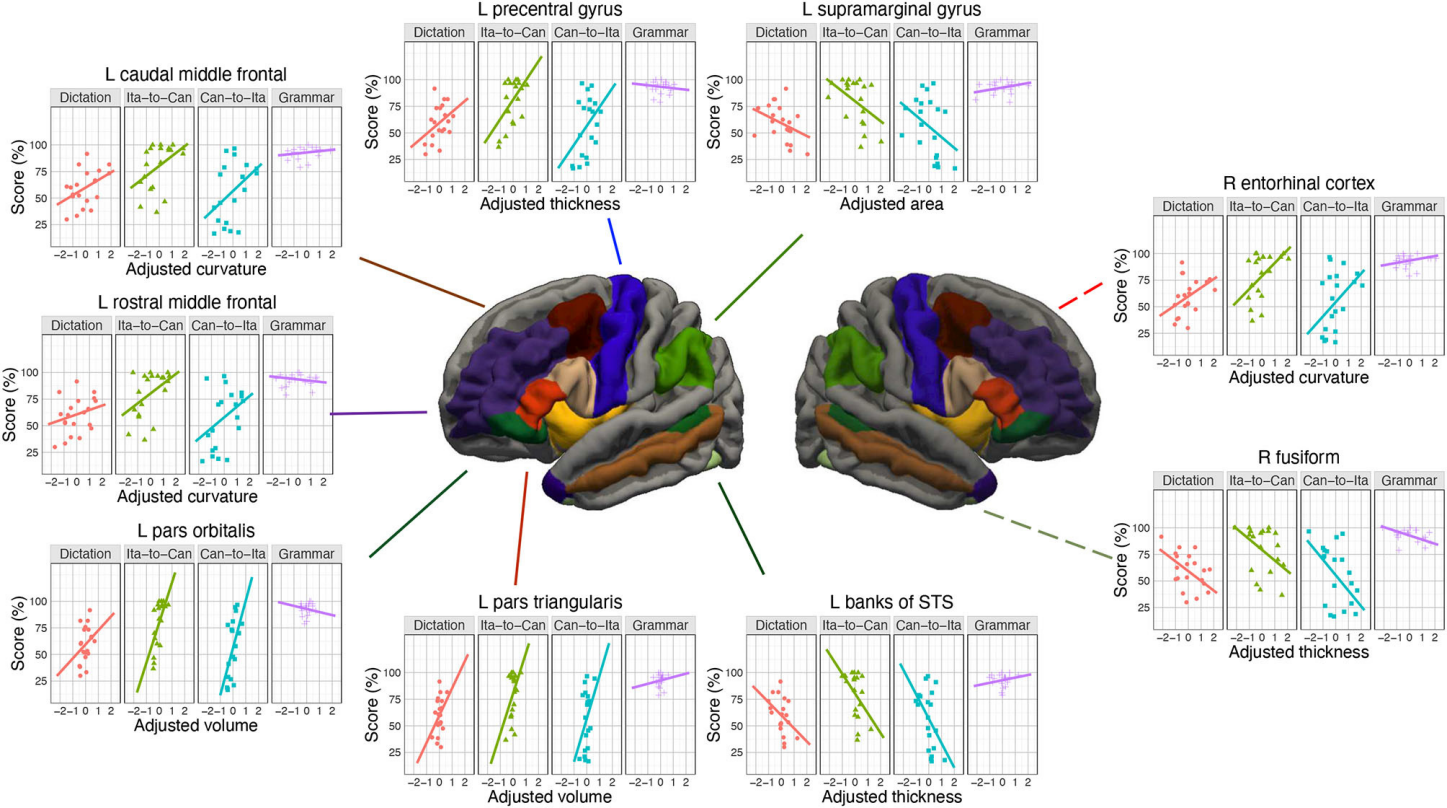
Associations of the final test score with the cortical measures. A more left-dominant network was implicated, with seven left hemisphere regions showing significant associations with the final test score when compared to only two right hemisphere regions.

For final test, significant associations of the test score with the cortical measures were found for nine ROIs. For (1) right entorhinal cortex, there was significant positive association between final test score and adjusted curvature, *t* = 3.06, *p* = 0.006, adjusted thickness, *t* = 2.56, *p* = 0.019, and adjusted area, *t* = 2.12, *p* = 0.047. TestPart × adjusted volume was significant, as the adjusted volume was negatively associated with only the two translation scores (Ita-to-Can, and Can-to-Ita). TestPart also interacted with adjusted curvature, which was positively associated with the first three parts (Dictation, Ita-to-Can, and Can-to-Ita), and adjusted thickness, which had the same association pattern except that its association with Dictation was only marginally significant. For (2) left pars orbitalis, the test score was significantly predicted by adjusted volume, *t* = 3.15, *p* = 0.005. The interaction between TestPart and adjusted volume was also significant, meaning that the association between the test score and adjusted area was modulated across different parts. For (3) left banks of STS, the test score was significantly and negatively associated with adjusted thickness, *t* = −2.63, *p* = 0.016. There were significant interactions of TestPart with adjusted thickness (which was significantly and negatively associated with translation scores) and adjusted area (which was marginally and negatively associated with both translation scores). For (4) right fusiform gyrus, the test score was significantly predicted by adjusted thickness, *t* = −4.59, *p* < 0.001. TestPart × adjusted thickness was significant: adjusted thickness was negatively associated with the scores in the first three parts. For (5) left SMG, the test score was positively associated with adjusted volume, *t* = 2.21, *p* = 0.039, and negatively associated with adjusted area, *t* = −2.33, *p* = 0.030. TestPart × adjusted area was also significant, with the negative association being significant for both translation scores. For (6) left precentral gyrus, TestPart interacted with both adjusted thickness and adjusted curvature, with the thickness being significantly associated with the two translation scores, while adjusted curvature was only significantly associated with Can-to-Ita. For (7) left pars triangularis, the test score was significantly predicted by adjusted volume, *t* = 2.52, *p* = 0.021. TestPart × adjusted volume and TestPart × adjusted curvature were significant, with both adjusted volume and curvature being positively contributing to the two translation scores. For (8) left caudal middle frontal cortex, the test score was significantly predicted by adjusted curvature, *t* = 3.16, *p* = 0.005. TestPart also interacted with adjusted curvature and adjusted area, with the adjusted curvature being positively associated with the test scores for the first three parts, and the adjusted area negatively associated with the two translation scores. For (9) left rostral middle frontal cortex, the test score was significantly and positively associated with adjusted volume, *t* = 2.19, *p* = 0.041, and with adjusted curvature, *t* = 2.11, *p* = 0.047. TestPart × adjusted curvature was significant, with the curvature being positively associated with the two translation scores. Marginal improvement over the baseline model was observed at one additional ROI (right SMG). For each ROI above, the representative interaction term was illustrated in Figure 5.

#### 3.2.3. Sub-cortical Volumetric Predictors of Learning Performance

The contributions of sub-cortical regions were also examined using sub-cortical volumes, normalized by the intracranial volume (ICV). None of the normalized sub-cortical volumes could significantly improve the two baseline models for the in-class vocabulary learning performances: Ita-to-Can, all uncorrected *p*'s >0.13, Can-to-Ita, all uncorrected *p*'s >0.16. For the final test score, the baseline model was significantly improved by two predictors, namely, the sub-cortical volume of left and right thalamus (Table 5). For left thalamus, the interaction TestPart × normalized volume was significant, *F*(3, 60) = 4.29, *p* = 0.008, due to the positive association of normalized volume with the scores in two parts: Ita-to-Can, *t* = 2.24, *p* = 0.031, and Can-to-Ita, *t* = 2.35, *p* = 0.024. For right thalamus, the interaction TestPart × normalized volume was significant, *F*(3, 60) = 3.74, *p* = 0.016, due to the significant positive associations of normalized volume with Can-to-Ita, *t* = 2.73, *p* = 0.009 and Ita-to-Can, *t* = 2.43, *p* = 0.019. There was only a weak indication for an association with the sub-cortical volume of right hippocampus (uncorrected *p* = 0.09).

**Table 5:** Sub-cortical ROIs showing significant associations with the final test scores.

**Region**	**AIC**	**BIC**	**logLik**	***r2m***	***r2c***	**χ^2^**	**Df**	**p (unc)**	**p (FDR)**	
L thalamus	669.78	700.75	−321.89	0.58	0.80	14.09	4	0.007	0.036	*
L caudate	683.15	714.11	−328.57	0.51	0.76	0.73	4	0.948	0.948	ns
L putamen	680.32	711.29	−327.16	0.54	0.77	3.55	4	0.470	0.626	ns
L hippocampus	679.81	710.78	−326.91	0.53	0.77	4.06	4	0.397	0.626	ns
R thalamus	670.33	701.30	−322.17	0.59	0.80	13.54	4	0.009	0.036	*
R caudate	682.64	713.60	−328.32	0.53	0.76	1.24	4	0.872	0.948	ns
R putamen	679.95	710.91	−326.97	0.54	0.77	3.93	4	0.416	0.626	ns
R hippocampus	675.83	706.80	-324.92	0.56	0.78	8.04	4	0.090	0.240	ns

*p(unc), uncorrected p, p(fdr), FDR-corrected p*.

*** *, p < 0.001*,

** *, p < 0.01*,

* *, p < 0.05*,

† *, marginal, ns, non-significant*.

### 3.3. Cognitive and Phonological Associations

#### 3.3.1. Correlations Among the Cognitive and Phonological Predictors

**Figure 6:**
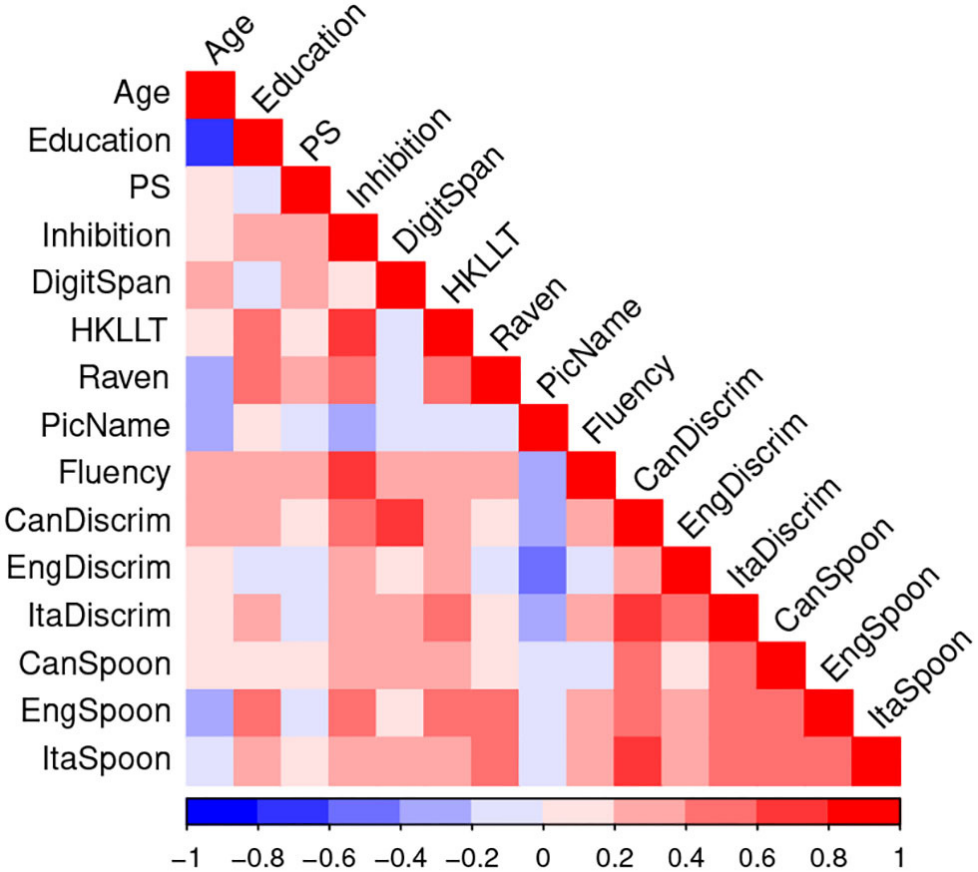
Correlation matrix among demographic, cognitive, and phonological measures at the initial-learning phase.

Figure 6 shows the correlations among the cognitive and phonological predictors. In general, there were high correlations among the three phonological discrimination scores (average *r* = 0.58, range = 0.45 – 0.76) and among the three Spoonerism scores (average *r* = 0.52, range = 0.47 – 0.56), but relatively low correlations across these measures. This pattern of correlation supported our a priori choice of selecting only one measure each of phonological discrimination and of Spoonerism for representing phonological skills. Given the non-phonological nature of the Chinese writing system, the majority of the older learners were unfamiliar with the phonetic system used in Cantonese. Consequently, the English phonological discrimination and Spoonerism scores were selected as the phonological predictors.

#### 3.3.2. Cognitive Models: Predictors of In-class and Final Test Performances

**Figure 7:**
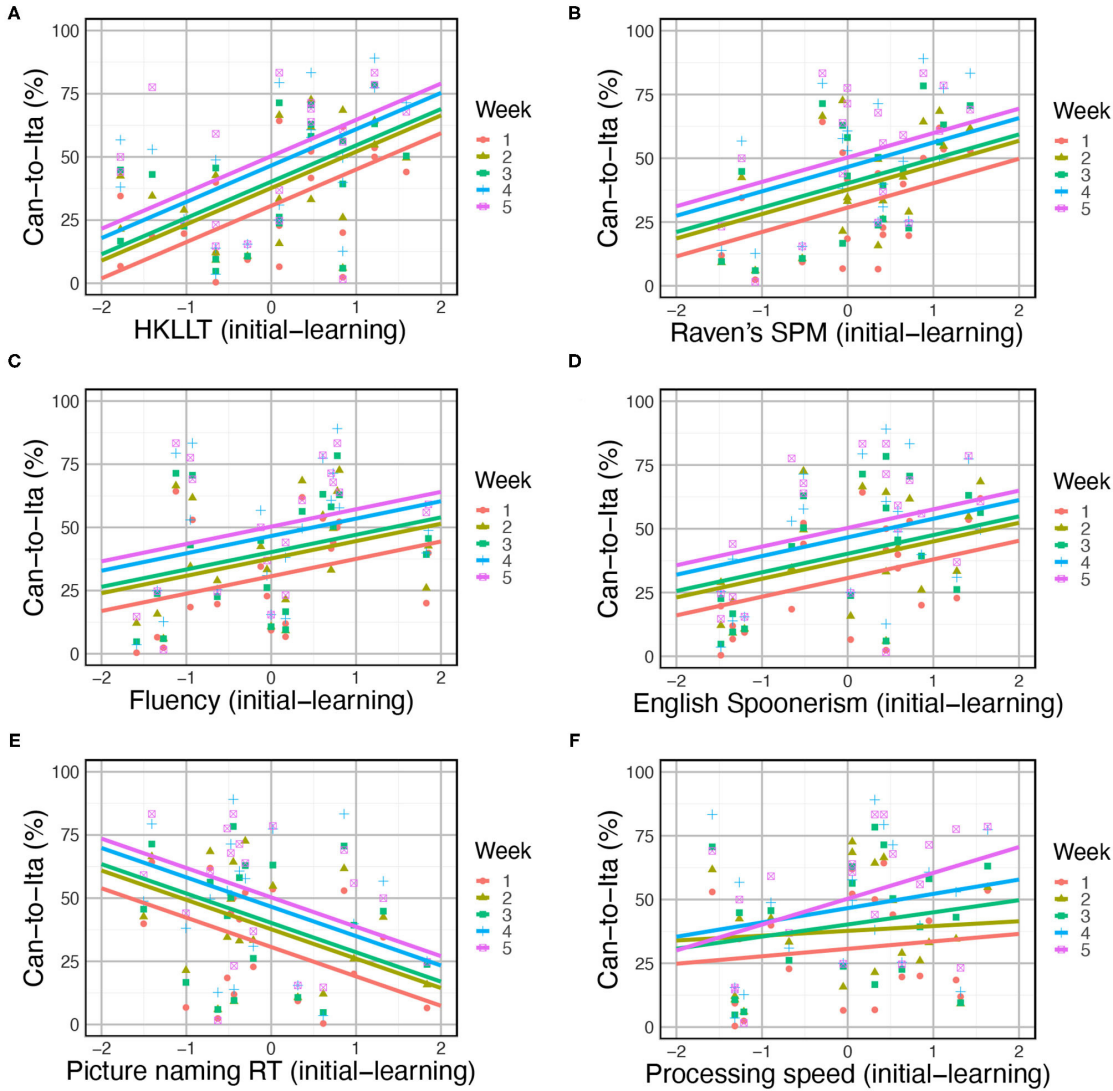
Modulation of in-class Can-to-Ita score by cognitive predictors, including **(A)** HKLLT, **(B)** Raven's SPM, **(C)** semantic fluency, **(D)** English Spoonerism, **(E)** picture naming, and **(F)** processing speed. Each point represents the score of one participant at a certain week, and the solid lines are the fitted data based on the LME models.

One of the three baseline models was significantly improved by the cognitive predictors: Can-to-Ita, *AIC* = 789.83, *BIC* = 933.11, *logLik* = −339.91, χ^2^ = 79.18, *DF* = 45, uncorrected *p* = 0.001, corrected *p* = 0.004. Backward elimination was applied to yield the final model (Table 6). The performance was positively associated with HKLLT (*t*(20) = 4.8, *p* < 0.001), Raven's SPM (*t*(20) = 4.33, *p* < 0.001), English Spoonerism (*t*(20) = 3.28, *p* = 0.004), semantic fluency (*t*(20) = 3.05, *p* = 0.006), and processing speed (*t*(20) = 2.49, *p* = 0.022), and as expected, negatively associated with picture naming latency (*t*(20) = −6.53, *p* < 0.001), but it was also negatively associated with digit span forward (*t*(20) = −6.34, *p* < 0.001) and inhibition (*t*(20) = −5.90, *p* < 0.001). The positive associations and the negative association with picture naming latency are illustrated in Figures 7A–E. In addition, Week × processing speed was also significant, *F*(4, 80) = 4.07, *p* = 0.005. This interaction effect was due to the general increase in the association of processing speed with the learning performance across time (Figure 7F), reaching statistical significance in Week 4 (*t*(20) = 2.26, *p* = 0.029) and Week 5 (*t*(20) = 4.09, *p* < 0.001).

**Table 6:** Cognitive models for the in-class Ita-to-Can and Can-to-Ita scores and for the final test, obtained after backward elimination of the full models.

**Score**	**Term**	**SumSq**	**MeanSq**	**NumDF**	**DenDF**	**F**	**p**	
Ita-to-Can ‡	Age	71.22	71.22	1	20.0	2.15	0.158	ns
	Gender	0.24	0.24	1	20.0	0.01	0.933	ns
	Education	2.23	2.23	1	20.0	0.07	0.798	ns
	Week	3156.37	789.09	4	80.0	23.87	<0.001	***
	HKLLT	75.30	75.30	1	20.0	2.28	0.147	ns
	Week:HKLLT	539.17	134.79	4	80.0	4.08	0.005	**
Can-to-Ita	Age	139.41	139.41	1	20.0	2.93	0.102	ns
	Gender	410.31	410.31	1	20.0	8.62	0.008	**
	Education	19.29	19.29	1	20.0	0.41	0.532	ns
	Week	4726.65	1181.66	4	80.0	24.84	<0.001	***
	PS	295.42	295.42	1	20.0	6.21	0.022	*
	Inhibition	1655.79	1655.79	1	20.0	34.80	<0.001	***
	DigitSpan	1912.07	1912.07	1	20.0	40.19	<0.001	***
	HKLLT	1095.99	1095.99	1	20.0	23.04	<0.001	***
	PicName	2028.22	2028.22	1	20.0	42.63	<0.001	***
	Fluency	441.17	441.17	1	20.0	9.27	0.006	**
	Raven	893.03	893.03	1	20.0	18.77	<0.001	***
	EngSpoon	511.33	511.33	1	20.0	10.75	0.004	**
	Week:PS	773.63	193.41	4	80.0	4.07	0.005	**
FinalTest ‡	Age	892.65	892.65	1	20.0	6.13	0.022	*
	Gender	478.92	478.92	1	20.0	3.29	0.085	†
	Education	6.57	6.57	1	20.0	0.05	0.834	ns
	TestPart	18082.02	6027.34	3	60.0	41.41	<0.001	***
	Fluency	693.43	693.43	1	20.0	4.76	0.041	*

‡ *The likelihood ratio test comparing the baseline and full model of the in-class Ita-to-Can score was non-significant after Bonferroni correction; the result tabulated here was obtained to explore the cognitive predictors of the in-class Ita-to-Can performance (see main text).*

*** *, p < 0.001*,

** *, p < 0.01*,

* *, p < 0.05*,

† *, marginal, ns, non-significant*.

For in-class Ita-to-Can, the improvement over the baseline model did not survive the correction, *AIC* = 774.01, *BIC* = 917.30, *logLik* = −332.01, χ^2^ = 62.72, *DF* = 45, uncorrected *p* = 0.041, corrected *p* = 0.124. The same held for the baseline model of the final test, *AIC* = 696.15, *BIC* = 803.34, *logLik* = −303.08, χ^2^ = 51.72, *DF* = 36, *p* = 0.043. However, backward elimination was applied to explore the most important predictor(s). For the in-class Ita-to-Can, the exploratory analysis revealed a significant Lesson × HKLLT interaction; the trend analysis revealed marginally significant positive associations in week 1 (*t*(80) = 2.05, *p* = 0.052) and week 3 (*t*(80) = 1.86, *p* = 0.076). For the final test, it was positively associated with semantic fluency (*t*(20) = 2.18, *p* = 0.041).

### 3.4. Cognitive Comparison Between the Initial-Learning vs. Post-learning Phase

Pairwise Wilcoxon signed rank tests revealed marginally shorter picture naming latency, *p* = 0.057, with an effect size (King et al., 2011) of *r*_*c*_ = −0.67, marginally better Raven score (*p* = 0.057, *r*_*c*_ = 0.67) and HKLLT (*p* = 0.094, *r*_*c*_ = 0.67). There were also marginally higher Italian discrimination score, *p* = 0.057, *r*_*c*_ = 0.67, as well as higher scores in the two Cantonese tests: CanDiscrim, *p* = 0.094, *r*_*c*_ = 0.57, and CanSpoon, *p* = 0.094, *r*_*c*_ = 0.62 (Table 7). However, due to the lack of a control group, it is unclear whether the change arose due to repetition effect or it was truly induced by intensive vocabulary learning.

**Table 7:** Comparisons of cognitive and phonological scores between initial- and post-learning phases.

**Test**	**Initial-learning**			**Post-learning**			***r_c_***	**p(unc)**	**p(FDR)**	
	**Mean**	**SD**	**Range**	**Mean**	**SD**	**Range**				
Cognitive										
PS	75.5	9.5	(60.5, 91)	76.3	9.7	(61, 93)	0.26	0.313	0.452	ns
Inhibition	0.46	0.09	(0.27, 0.60)	0.46	0.06	(0.36, 0.57)	−0.10	0.729	0.729	ns
DigitSpan	9.1	1.6	(5.5, 11.5)	9.2	1.3	(7.5, 12.5)	0.19	0.571	0.710	ns
HKLLT	11.8	2.7	(7, 16)	12.8	2.6	(8, 16)	0.67	0.043	0.094	†
Raven	0.67	0.97	(−1.89, 2.05)	1.22	0.95	(−0.98, 2.40)	0.67	0.009	0.057	†
PicName	1358.7	221	(1026.4, 1821.9)	1247	276.2	(853.8, 1946.9)	−0.67	0.007	0.057	†
Fluency	12.6	2.6	(8.5, 17.3)	12.7	2.6	(8.3, 16.8)	0.14	0.601	0.710	ns
Phonological										
CanDiscrim	19.6	7.6	(7, 35)	22.3	6.7	(11, 39)	0.57	0.036	0.094	†
EngDiscrim	21	5.3	(8, 29)	23.2	5.9	(5, 29)	0.42	0.111	0.206	ns
ItaDiscrim	14.9	4.9	(6, 25)	16.9	5.4	(6, 26)	0.67	0.013	0.057	†
CanSpoon	4.1	6.4	(0, 23)	7.5	8.5	(0, 28)	0.62	0.031	0.094	†
EngSpoon	10.8	7.3	(0, 22)	11.8	7.4	(0, 24)	0.12	0.685	0.729	ns
ItaSpoon	5.2	5.5	(0, 19)	6	6.9	(0, 24)	0.32	0.224	0.364	ns

*r_c_, effect size of the Wilcoxon signed rank test, p(unc), uncorrected p, p(FDR), FDR-corrected p*.

*** *, p < 0.001*,

** *, p < 0.01*,

* *, p < 0.05*,

† *, marginal, ns, non-significant*.

## 4. Discussion

### 4.1. Semantic, Episodic, and Phonological Associations With Vocabulary Learning

Taking an individual-differences approach, the present study used both anatomical and cognitive measurements to investigate the basis of FLL in older adults, focusing on whether semantic and episodic memory play an especially important role in vocabulary learning within this special group of language learners.

In support of the hypothesis that semantic functions are important for older language learners, the left pars orbitalis, which is central to semantic control (Sabb et al., 2007; Binder et al., 2009; Ralph et al., 2017) by virtue of its white matter pathway (uncinate fasciculus) to the semantic hub in the temporal pole (Harvey et al., 2013), was found to be associated with the performance in the in-class quiz and the final test. In particular, its adjusted curvature was positively associated with the in-class Can-to-Ita performance while its adjusted volume was significantly associated with the first three parts of the final test (dictation, Ita-to-Can, and Can-to-Ita). This suggests that high-performing learners tend to have higher adjusted curvature and volume in left pars orbitalis. To our knowledge, the importance of the left pars orbitalis has seldom been reported in previous works in which the learner group comprised mostly younger adults, even though a whole-brain analysis was conducted in these studies (e.g., Bellander et al., 2016). Instead, it is generally understood that the anatomical measures of the left IFG, as the seat of the Broca's area, are sensitive to language processes and bilingualism. For example, its gray matter volume was positively correlated with L2 performance (Mårtensson et al., 2012; Stein et al., 2012). Its cortical thickness was also larger for bilinguals that acquired their L2 in late childhood (8–13 years) than in early childhood (4–7 years) (Klein et al., 2014). Our finding is consistent with this general understanding, but further suggests that the pars orbitalis is especially associated with both immediate and long-term vocabulary retention. It is worth noting that the left temporal pole has barely failed to reach statistical significance in predicting in-class Can-to-Ita (FDR-corrected *p* = 0.053), which would have provided another piece of convergent evidence.

In support of the hypothesis that episodic memory is also important for older language learners, associations with the performance of both the in-class quiz and the final test were found at the left caudal middle frontal cortex. In particular, the mean curvature of the left caudal frontal cortex was consistently positively associated with the learning performance, including the in-class Can-to-Ita score regardless of week and the first three parts of the final test. By comparison, the adjusted thickness only significantly predicted the in-class Can-to-Ita over Weeks 1–3 while the adjusted cortical surface area was negatively associated with the two translation scores in the final test. In the literature, the left caudal middle frontal cortex is known to be associated with semantic strategies (Kirchhoff et al., 2014; Yu et al., 2018), referential encoding strategies (Yang et al., 2013), self-initiated elaborative encoding strategies (Husa et al., 2017), and working memory (Petrides et al., 1993). The present finding suggests that better-performing older learners might have employed a variety of encoding strategies in vocabulary learning. The present result corroborated with two previous works. Yang et al. (2015) reported that the middle frontal cortex was more active in successful learners in a tone discrimination task. The authors suggested that the higher level of activation was associated with the path between middle frontal gyrus and inferior parietal lobe, where lexical knowledge was automatically activated and sent to middle frontal gyrus. Another study found that the cortical thickness of MFG was increased after FLL (Legault et al., 2019b). Apart from the left caudal middle frontal cortex, converging evidence was also found in the left entorhinal cortex, which is implicated in the episodic processing of object/event information in relation to the context (Eichenbaum et al., 2012). Specifically, the in-class Can-to-Ita performance was positively associated with its cortical thickness.

In contrast, for regions that are especially important for phonological processing, they were associated with only long-term retention. Specifically, the left precentral gyrus is important for phonological working memory, serving as a locus for phonological rehearsal (Veroude et al., 2010; Novén et al., 2019), while the left SMG is known to play important role in phonological processing skills (Veroude et al., 2010; Sliwinska et al., 2012). In partial support to the hypothesis that phonological skills play a role in learning success, for left precentral gyrus, its adjusted thickness and curvature were significantly or marginally associated with the two translation tasks in the final test. The adjusted volume of left SMG was positively associated with the final test score in general, while the adjusted area was negatively associated with the scores in the two translation scores of the final test. The lack of associations of in-class scores with the precentral gyrus and left SMG associations, coupled with the presence of such associations with left pars orbitalis and left caudal middle frontal gyrus, is consistent with our hypothesis that the semantic and episodic memory functions play a more important role for FLL in older adults.

We speculate that semantic and episodic memory processes would be especially needed for intensive vocabulary learning; they are involved, for example, in strategically and proactively generating idiosyncratic semantics-based mnemonics (Thomas and Wang, 1996; Khoii and Sharififar, 2013) or referential encoding strategies for the new words (Turk et al., 2015). Suppose the learner is not confident that they can directly retrieve the meaning of *la mela* (“the apple,”) a possible cue for retrieval could be the semantic category “fruit,” which could in turn be encoded in terms of the learner's native language. Referential encoding strategies may also be relevant, as the learner could encode the contextual information, e.g., the noun “la mela” was learnt within the word list associated with the verb *mangiare* (“to eat”) or it was the first word in the first list. Having such cues facilitates the word recall during the in-class translation quizzes. Learners who used these cues may also tend to have better performance in long-term retention, even though the learners no longer need such strategies after many weeks of consolidation.

The cognitive associations reported are consistent with the putative reasons for the strong association of the FLL performance with the left pars orbitalis and left caudal middle frontal cortex discussed above. Across the three cognitive models, only semantic fluency and the delayed recall in the Hong Kong List Learning Test (HKLLT), as measures of semantic retrieval and verbal episodic memory functions, were significant predictors in more than one final models. Specifically, HKLLT significantly contributed to the two in-class scores but not the final test; this pattern suggested that verbal episodic memory is more associated with the immediate learning than long-term language retention performance. In contrast, semantic fluency significantly predicted both in-class Can-to-Ita and the final test. Thus, the evidence suggested that semantic fluency was associated with both immediate learning and long-term retention. One reason that semantic fluency was not significant for predicting in-class Ita-to-Can is that controlled semantic retrieval ability, as captured by the semantic fluency task, has a strong association with the use of semantic associations and strategies. During vocabulary learning, most learners tend to be able to make a direct association of the L2 (Italian) word with the L1 (Cantonese) word, as reflected by a relatively high performance of the Ita-to-Can task in the present study. In contrast, they were much less accurate in the Can-to-Ita task, indicating that they had difficulty in recalling the same association in the reverse direction, i.e., from the L1 (Cantonese) word to the L2 (Italian) word. Such asymmetry has been a point of emphasis in modeling the acquisition of foreign vocabulary, e.g., the parasitic model of vocabulary acquisition (Ecke, 2015). Due to the relative difficulty in Can-to-Ita, the task is subject to a greater use of mnemonic strategies for retrieval. The stronger association of semantic fluency with Can-to-Ita than with Ita-to-Can suggests that semantic retrieval strategies could have been utilized more in the Can-to-Ita task.

Besides HKLLT and semantic fluency, the remaining cognitive measures were kept in at most only one model, namely, in-class Ita-to-Can. In addition to processing speed, which is known to be a general deciding factor of the performance of older adults across domains (Salthouse, 1996), the in-class Ita-to-Can was associated with faster picture naming latency and better Raven's SPM. In contrast, in terms of phonological function measures, while EngSpoon contributed positively to in-class Can-to-Ita, the opposite was true for digit span forward (an index of phonological STM), suggesting that phonological function measures do not necessarily contribute positively. The inhibition function, measured using Stroop Color and Word Test, was also negatively to the learning performance.

Taken together, the anatomical and cognitive models of the learning performance were consistent with our main hypothesis that both semantic and episodic memory functions are prominently implicated in older adults. Phonological skills were also implicated, but their associations with learning performance were not as consistently observed.

### 4.2. Differential Hemispheric Associations in Immediate and Long-Term Retention

Beyond our main hypothesis, the present results could also be viewed from another perspective, in that there were interesting differences in the global pattern of anatomical associations between immediate learning and long-term retention (see Figures 4, 5). As discussed above, the left pars orbitalis and left caudal middle frontal cortex were the only two regions that consistently showed associations with the performance in both immediate learning (Can-to-Ita) and long-term retention. All other regions were only associated with the performance of either the in-class Can-to-Ita or the final test score.

For the in-class Can-to-Ita performance, there were five such regions: left entorhinal cortex, right insula, right pars orbitalis, right caudal anterior cingulate, right middle temporal gyrus. Among them, significant anatomical associations with Can-to-Ita performance were found at left entorhinal cortex (see section 4.1) and right insula. The insula is known to be involved in procedural memory and rule-based learning (Ullman, 2001a, 2004; Yang and Li, 2012). In the present study, the in-class Can-to-Ita score was positively associated with the adjusted volume of the right insula but negatively associated with its adjusted surface area and curvature. In contrast to the right insula, the remaining three right hemisphere regions only showed some subtle changes in the association strength with the in-class Can-to-Ita score, in the sense that while the association was non-significant in any of the five weeks, the magnitude of such association varied significantly across weeks. Taken together, the significant association of right insula and the significant modulation of association over the other three right hemisphere regions suggest that the right hemisphere is implicated in the initial-learning phase. Such right hemisphere associations above added to the literature that reported some associations of the right hemisphere with FLL performance (Hosoda et al., 2013) or bilingualism-induced neuroplasticity (Bubbico et al., 2019; Maschio et al., 2019; Legault et al., 2019b), although the precise roles should be tested using online functional MRI tasks.

For long-term language retention, beyond left pars orbitalis and left caudal middle frontal cortex, seven regions were additionally associated with the final test results: left precentral gyrus (see section 4.1), left SMG (see section 4.1), left pars triangularis, left rostral middle frontal cortex, left banks of superior temporal sulcus, right fusiform gyrus, and right entorhinal cortex. In other words, a more left-dominant network was implicated in long-term vocabulary retention, with seven left hemisphere regions showing significant associations with the final test score when compared to only two right hemisphere regions. Also, unlike the subtle right-hemispheric associations with in-class Can-to-Ita, all regions here showed significant associations with individual test scores. For the left hemisphere, the inclusion of left pars triangularis was not surprising, given that it is a part of the Broca's area. The left rostral middle frontal cortex has been implicated in language learning (Sheppard et al., 2012; Novén et al., 2019), while the left banks of superior temporal sulcus is known to reflect verbal intelligence and receptive language functions in children (Li et al., 2020). For the right hemisphere, the right fusiform gyrus is well-known for its role in object recognition (as opposed to word recognition for the left fusiform gyrus), while the right entorhinal cortex was especially important for long-term memory consolidation (Haist et al., 2001; Piefke et al., 2003).

Overall, while the best anatomical predictor of immediate success was found on semantics-associated (the left pars orbitalis) and episodic memory-related regions (left caudal middle frontal gyrus and the left entorhinal cortex), there were significant association with the right insula and some subtle changes in association with the right pars orbitalis, right rostral anterior cingulate, and right middle temporal gyrus over the course of learning. The increased left-lateralization in the association pattern of the final test scores was consistent with the view that the right-hemisphere homologs of the left-hemisphere language areas may provide “scaffolding” in the early phase of FLL, before a right-to-left shift occurs (Yang et al., 2015; Qi and Legault, 2020).

### 4.3. The Anatomical Associations of Sub-cortical Regions

In previous works, increased hippocampal volume was observed in Swedish individuals studying Italian (Bellander et al., 2016). The hippocampal volume was also a good predictor of the achieved vocabulary proficiency (Nilsson et al., 2021). Apart from the hippocampus, the striatum has been highlighted in previous works (Ullman, 2004; Abutalebi and Green, 2007; Li et al., 2014; Legault et al., 2019a). However, instead of the hippocampus and striatum, our results showed that the thalamus has stronger association with long-term vocabulary retention, with the cortical volumes of the bilateral thalami showing positive associations with the final test scores. The thalamus is implicated in language production by selecting lexical and semantic representations (Abutalebi and Green, 2016), and an expansion of thalamus is associated with greater second language immersion (Pliatsikas et al., 2017; Deluca et al., 2019a). The present finding added to the literature showing that the structural parameters of thalamus are sensitive to aspects of FLL, although the underlying reasons for the associations should be investigated further.

For hippocampus, we observed some degree of association of the right hippocampal volume with the Can-to-Ita test score, but the association did not survive the FDR correction. Indeed, while the role of hippocampus in associative memory, especially in various learning and recall paradigms, is well-established in patient populations in the neurological literature (Suzuki, 2008), direct associations of hippocampal volume with associative memory have not been consistently found in cognitively normal older adults (Becker et al., 2015; Zheng et al., 2017). Finally, for striatum, the sub-cortical volumes were significantly associated with none of the three learning measures. One possibility is that these structures are non-unitary in functions. A finer-grained atlas may be necessary to reveal the associations.

### 4.4. Conclusion and Limitations

Previous studies have suggested that structural associations could provide valuable information for inferring functional associations (Kanai and Rees, 2011; Chiarello et al., 2013). In the present study, significant associations with vocabulary learning performances were consistently found for the left pars orbitalis and left caudal middle frontal cortex. These results suggest that the individual variations in structural morphometry of the prefrontal lobe are strongly associated with language learning success in older adults, considering that the prefrontal lobe is known to be involved in many “higher” cognitive skills such as language, reasoning, and planning (Wood and Grafman, 2003). In particular, the structural brain and cognitive models were consistent with our main hypothesis that semantic and episodic memory functions likely play an important role in language learning in older adults, with the regions most implicated with these functions being prominently represented in the overall pattern of associations. It should be noted that the present study had not explicitly tested what cognitive processes were involved during learning, given that an online design was not employed. Indeed, an alternative account for the association pattern observed is that the surface-based morphological features and the cognitive predictors are good indices of language aptitude, as previous studies suggested (e.g., Novén et al., 2019). Which could be a parsimonious and alternative explanation why these measures could significantly predict the FLL performance. Nonetheless, language aptitude also has multiple components that are differentially associated with cognitive functions, which could provide a more domain-general account of the anatomical associations with learning performance.

Our research complemented previous works that put a stronger focus on the acquisition of other linguistic elements such as phonology (Wong and Perrachione, 2007) and grammar (Yang and Li, 2012). However, due to the absence of a young learner group in the present study, no conclusion could be drawn regarding the relative contributions of semantic functions in FLL across older and younger adults. On the other hand, the majority of the studies thus far were on younger learners, and the role of the left pars orbitalis and left caudal middle frontal gyrus have seldom been reported. In this respect, the present findings suggest that semantic functions could play a more important role in older adults, although this hypothesis should be re-examined with online experimental designs.

Also, a strength of the present study was that objective measures of learning performance were collected throughout the Italian vocabulary learning protocol. Such longitudinal data play a crucial role in the sensitivity of the present analysis in revealing the anatomical markers of learning performance, despite our relatively modest sample size. In addition, by incorporating different surface-based measures into our univariate LME models, we successfully revealed a relatively consistent pattern of significant brain–learning associations. Where an association was found, the adjusted cortical volume was consistently a positive predictor of learning performance, with only one exception found at the fusiform gyrus. Similarly, the adjusted curvature of the regions involved was a strong positive predictor of learning performance, with only two exceptions found for the left entorhinal cortex and the right insula. In contrast, the adjusted area was consistently a negative predictor of the learning performance, without any exception. Adjusted thickness showed the least consistent pattern, being a positive predictor at four ROIs but a negative predictor at two ROIs.

The present work was limited in several ways. First, the MRI scans were not acquired at the beginning of the Italian learning programme but after an introductory Italian lesson of 90 min, to acclimate the learners with a more uniform experience with the target language prior to the scanning. Previous studies have shown that measurable changes in diffusion MRI indices could be induced by short-term intervention of 45 min (Tavor et al., 2020) or 120 min (Sagi et al., 2012). Structural changes were found after 20 days of language learning (Legault et al., 2019b), with some even estimating that only 2 h of training could bring about structural changes in the brain (Park and Bischof, 2013). However, considering that such structural changes may not be long-lasting, and the neuroplasticity of older adults' brains are lower than younger adults' (e.g., Freitas et al., 2011), it is unlikely that a 1.5-h lesson could significantly influence the anatomical measures extracted, to a point that their associations with learning performance would change qualitatively. Secondly, we acknowledge that from the experimenter's perspective, it would have been best to arrange the cognitive and phonological tests prior to the introductory Italian lesson. However, at the beginning of the programme, most participants were anxious and most motivated to start learning Italian. As such, arranging the prolonged testing of about 3.5 h that are not directly related to learning Italian could be seen as a non-ideal arrangement from the learner's perspective, which could lower their engagement in the programme, leading to an increased dropout rate. Because it was not our primary goal to investigate the learning-induced cognitive gains, a compromise was made to spread the cognitive and phonological tests over visits 3 to 6. Considering that a cognitive gain or an improvement of phonological skills could have already incurred following the 90-min introductory lesson and the 2-h vocabulary lessons in the initial-learning phase, we acknowledge that our decision to delay these assessments could have led to an underestimation of the cognition gains or phonological skill improvements reported. Finally, no post-learning MRI scan was conducted, such that any learning-related neuroplasticity could not be tested. The lack of a younger group also precluded any formal hypothesis testing on the differential brain–learning associations between young and older learners. These issues will be addressed in our next round of data collection.

## Data Availability Statement

The raw data supporting the conclusions of this article will be made available by the authors, without undue reservation.

## Ethics Statement

The studies involving human participants were reviewed and approved by Institutional Review Board, The Hong Kong Polytechnic University. The patients/participants provided their written informed consent to participate in this study.

## Author Contributions

MF, AA, and WW contributed to the conception and grant application of the study. MF and TL implemented the Italian learning protocol in the present study. MF, JC, TL, MM, and N-YH were involved in data collection and data transcription. MF and MM analyzed the data and wrote the manuscript. MF, MM, and WW contributed to the interpretation of the results. All authors contributed to the article and approved the submitted version.

## Funding

This research was partially supported by HKRGC-GRF grant 15606119 and Dean's Reserve from the Faculty of Humanities, The Hong Kong Polytechnic University, both awarded to WW. The article processing charge was supported by the Research Institute for Smart Aging (RISA), The Hong Kong Polytechnic University.

## Conflict of Interest

The authors declare that the research was conducted in the absence of any commercial or financial relationships that could be construed as a potential conflict of interest.

## Publisher's Note

All claims expressed in this article are solely those of the authors and do not necessarily represent those of their affiliated organizations, or those of the publisher, the editors and the reviewers. Any product that may be evaluated in this article, or claim that may be made by its manufacturer, is not guaranteed or endorsed by the publisher.
