# Supplementary material for: Foreign Language Learning in Older Adults: Anatomical and Cognitive Markers of Vocabulary Learning Success
Source: Front Hum Neurosci. 2022 Mar 7;16:787413. doi: 10.3389/fnhum.2022.787413 (PMC8942782; doi:10.3389/fnhum.2022.787413)
Supplement: Supplementary file 1 [file Data_Sheet_1.pdf]

Supplementary Material

S1 ITALIAN VOCABULARY LEARNING PROGRAMME

S1.1 Italian vocabulary lessons

Table S1 shows the complete set of 60 Italian word lists used in the learning programme. Each list comprised a transitive verb (e.g., *mangiare*, “to eat”) and six matching nouns (e.g., *la mela*, “an apple”). In constructing these word lists, control measures were taken so that any systematic changes in the learning performance over time could not be attributed to confounding factors like the inherent difficulties of the Italian word lists or their Cantonese counterparts. In particular, because Italian words with a larger number of syllables or letters would likely be more difficult to remember, 5 groups of 12 word lists were constructed, with each group being the study materials for two consecutive lessons. Across all pairs of groups, the verbs and nouns were matched in both mean number of syllables (verbs: ps >0.14, nouns: ps >0.10) and mean number of letters (verbs: ps >0.32; nouns: ps >0.14). The word lists were also matched in their Cantonese translation, in terms of the mean number of Chinese characters per word (verbs: ps >0.10; nouns: ps >0.07) and mean number of strokes per word (verbs: ps >0.28; nouns: ps >0.12). See the supplementary data (`Table 1.XLSX`) for the Cantonese and English translation. The stimulus matching was not conducted by lesson, because, due to the non-arbitrary pairing of the verbs and nouns, it was exceedingly difficult to create 10 groups that were matched in all four variables. Dividing by week also provided a better match in terms of the structure of the whole programme, in that the majority of older adults had two lessons per week, and a revision session was arranged every two lessons starting the third vocabulary lesson. By using week rather than lesson as a measurement unit, each in-class score was the average over two lessons, which should have lower measurement noise compared to that derived using only one lesson. The auditory files for the Italian words were downloaded from https://ttsmp3.com/text-to-speech/Italian/ (using “Carla” and “Giorgio” for female and male voices, respectively). Within each lesson, the two voices were used in alternating order across the six word lists. The teaching materials in each lesson were delivered based on a custom-made E-Prime 3.0 script.

S1.2 Italian final test

The 1.5-h Italian final test was administered post-learning, and it comprised four different parts: dictation, Ita-to-Can, Can-to-Ita translation, and grammar. **Table S2** shows the vocabulary tested in the first three parts of the final test (dictation, Ita-to-Can, and Can-to-Ita). In these parts, the words were uniformly sampled across the ten vocabulary lessons. For the grammar test, the questions were multiple-choice questions with either two or four choices. Each such question was worth two or four marks, respectively. The distribution of points approximately matched the relative emphasis in the grammatical notes; out of a maximum of 180 points, gender, singular and plural forms, article, present tense, adjective, numeral, and possessive adjective each accounted for 20, 20, 20, 40, 40, 20, and 20 marks.

S1.3 Debriefing session

During the debriefing session, for educational purpose, all participants were given a simple written report summarizing their basic brain measures (e.g., intracranial volume, volume of lateral ventricles), cognitive performance, and Italian learning performance. They would also fill in some questionnaires and other short tests.

Table S1. The complete set of 60 Italian word lists used in the Italian learning programme. Each word list (1-60) comprised a verb (V) and six corresponding nouns (N1-N6).

List	V	N1	N2	N3	N4	N5	N6
1	mangiare	il cocomero	la mela	le ciliegie	le uve	la pesca	le fragole
2	condividere	un'insalata	una zuppa	il pane	un tramezzino	una bistecca	una torta
3	mancare	il padre	la madre	il fratello	la sorella	il figlio	la figlia
4	amare	i gatti	i cani	i conigli	i soldi	l'oro	l'argento
5	odiare	le formiche	le libellule	le mosche	le rane	le zanzare	i ragni
6	lavare	le mani	i piedi	gli orecchi	i capelli	la faccia	la schiena
7	portare	un asciugamano	contanti	un biglietto	una valigia	uno zaino	un cesto
8	prenotare	una barca	un treno	un volo	un posto	un albergo	un ostello
9	noleggiare	una macchina	una bicicletta	una motocicletta	un autista	una stanza	un attrezzo
10	toccare	il lupo	la volpe	l'orso	il pappagallo	il gufo	il delfino
11	pianificare	un giorno	una settimana	un mese	un anno	una mattina	un pomeriggio
12	pagare	la tassa	l'affitto	la bolletta	l'imposta	il prestito	la multa
13	incontrare	il nonno	la nonna	la nipote	lo zio	la zia	il coltivatore
14	assumere	un ragioniere	un cameriere	un tuttofare	un pittore	un cantante	un allenatore
15	volere	una gonna	un grembiule	le scarpe	le pantofole	gli occhiali	un distintivo
16	prendere	una cintura	una manetta	un anello	una spilla	una forcina	un fazzoletto
17	cucinare	il riso	il pollo	il pesce	l'aragosta	i capasanti	i gamberetti
18	cuocere	un uovo	il manzo	le vongole	il cavolo	i fungi	le verdure
19	bere	l'acqua	il latte	il frullato	il succo	la birra	il vino
20	tagliare	il cetriolo	il pomodoro	la zucca	la cipolla	lo zenzero	l'aglio
21	leggere	il romanzo	i fumetti	la rivista	le notizie	la pubblicità	l'orario
22	scrivere	un libro	una fiaba	un saggio	una cartolina	una ricetta	un elenco
23	costruire	una scuola	un ospedale	una casa	una fabbrica	una chiesa	una banchina
24	lasciare	l'aula	la biblioteca	la piscina	il benzinaio	il mercato	il negozio
25	indossare	un abito	una camicia	pantaloni	un pigiama	stivali	calzini
26	togliere	il cappotto	la sciarpa	la cravatta	il cappello	la paglietta	i guanti
27	ascoltare	il suono	la cazone	il campanello	la domanda	il discorso	la risata
28	dire	un racconto	uno scherzo	un indovinello	la risposta	la verità	una bugia

29	disegnare	il prato	la collina	la spiaggia	il lago	la cascata	le onde
30	imparare	una materia	la storia	la lingua	la maglieria	la ceramica	il gioco
31	spegnere	il formello	il forno	il riscaldatore	lo schermo	l’altoparlante	la stampante
32	prestare	la cancelleria	una matita	una gomma	una penna	un bianchetto	una riga
33	aprire	gli occhi	la bocca	la busta	la scatola	il sacchetto	la bottiglia
34	chiudere	il cancello	la finestra	la tenda	la guardaroba	la credenza	il cassetto
35	guardare	il cielo	le nuvole	le stelle	la neve	l’arcobaleno	il tramonto
36	sentire	l’erba	il mare	l’albero	il legno	la spezia	il fumo
37	dare	un pallone	una colla	una coperta	un secchio	una scala	un assegno
38	spedire	un fiore	un regalo	un pacchetto	una richiesta	un avvertimento	una scusa
39	perdere	un bastone	una racchetta	un lavoro	la sicurezza	la fiducia	la speranza
40	cercare	la bussola	la chiave	il telecomando	la vite	il fiume	aiuto
41	nascondere	le forbici	la freccia	la pallottola	la corda	la sega	la spada
42	avere	i baffi	le rughe	una cicatrice	un sorriso	un segreto	un premio
43	cambiare	il lenzuolo	la lampadina	lo pneumatico	l’acconciatura	l’indirizzo	l’abitudine
44	pulire	il soggiorno	la dispensa	il bagno	la vasca	il tavolo	il tappeto
45	nutrire	la mucca	il cavallo	la scimmia	il maiale	la pecora	il passero
46	catturare	un’ape	un uccello	una farfalla	un cervo	un topo	un serpente
47	fare	un orologio	una bambola	un aquilone	un astuccio	una cornice	una sedia
48	raccogliere	giocattoli	monete	accendini	conchiglie	francobolli	foglie
49	comprare	una forchetta	un coltello	un cucchiaio	una padella	una scopa	un mocio
50	vendere	frigoriferi	aspirapolvere	cellulari	affreschi	artigianati	antiquariati
51	gettare	la caramella	il gesso	il cuscino	la pietra	il ramo	l’immondizia
52	rompere	la tazza	la ciotola	il piatto	lo specchio	il vetro	la serratura
53	temere	il tuono	il fulmine	i temporali	i terremoti	i ladri	i vermi
54	evitare	il pericolo	le siccità	le frane	le malattie	le carestie	le guerre
55	iniziare	la colazione	il pranzo	la cena	il viaggio	lo spettacolo	l’indagine
56	tenere	una riunione	una mostra	un matrimonio	un banchetto	una festa	una fiera
57	essere	un amico	un ragazzo	una ragazza	un rivale	un re	una regina
58	diventare	un pompiere	un’infermiera	un insegnante	un marito	una moglie	un nemico
59	indicare	il labbro	il collo	il petto	la vita	la pancia	il ginocchio
60	bendare	la testa	la spalla	il braccio	il dito	la gamba	la ferita

Table S2. Vocabulary tested in the first three parts of the final test (dictation, Ita-to-Can, and Can-to-Ita). The stimuli for each part were uniformly sampled across the 10 vocabulary lessons. This table lists the questions for dictation and Ita-to-Can, and the answers for Can-to-Ita.

Dictation		Ita-to-Can	Can-to-Ita
le ciliegie	il riscaldatore	condividere una bistecca	mangiare la mela
un tramezzino	una riga	mancare la madre	amare i conigli
il fratello	la bocca	lavare la schiena	odiare le zanzare
l'argento	il cassetto	portare un asciugamano	prenotare un treno
le rane	il tramonto	toccare il gufo	noleggiare un attrezzo
i capelli	il legno	pagare l'affitto	pianificare un pomeriggio
una valigia	una colla	incontrare lo zio	assumere un ragioniere
un posto	un avvertimento	prendere un fazzoletto	volere un grembiule
una bicicletta	un lavoro	cucinare i capasanti	cuocere il cavolo
l'orso	la bussola	tagliare la zucca	bere il vino
una mattina	la corda	scrivere un saggio	leggere i fumetti
l'imposta	un segreto	lasciare il benzinaio	costruire una chiesa
la nipote	il lenzuolo	indossare una camicia	togliere la sciarpa
un cameriere	la dispensa	dire la risposta	ascoltare la domanda
le scarpe	il cavallo	disegnare il lago	imparare la ceramica
un anello	un uccello	spegnere la stampante	prestare una matita
l'aragosta	una sedia	chiudere il cancello	aprire la scatola
i fungi	accendini	sentire il mare	guardare le stelle
il latte	una padella	dare un pallone	spedire una scusa
il pomodoro	antiquariati	perdere una racchetta	cercare il telecomando
la rivista	il gesso	avere i baffi	nascondere la sega
una fiaba	la tazza	pulire il tappeto	cambiare l'acconciatura
un ospedale	il tuono	catturare un topo	nutrire il passero
la biblioteca	il pericolo	fare un astuccio	raccogliere foglie
un abito	la colazione	comprare una forchetta	vendere aspirapolvere
la paglietta	una riunione	gettare il cuscino	rompere il vetro
il discorso	un ragazzo	temere i vermi	evitare le carestie
un racconto	un marito	iniziare il pranzo	tenere un matrimonio
la cascata	il ginocchio	diventare un'infermiera	essere un re
la lingua	la gamba	bendare la ferita	indicare il petto

S2 COGNITIVE BATTERY

S2.1 Stroop color and word test

This test comprised three sub-tasks: word naming, color naming, and Stroop color–word naming, and was adapted from a standard test in English (Golden and Freshwater, 1978) to Chinese in our previous works (Fong et al., 2020, 2021; Hui et al., 2020). In all sub-tasks, 120 stimuli were arranged in six columns of twenty stimuli each on a computer monitor. In the first sub-task, each stimulus was one of the four color words (red, yellow, green, and blue), printed in Chinese (font: DFKai-SB) and in a white font color against a black background. In the second sub-task, each stimulus comprised four X's arranged in the four quadrants of a square (simulating the square shape of Chinese characters); the stimuli were printed in the four colors above. In the third sub-task, each stimulus was one of four color words, but each was printed in a color incongruent with the meaning of the word. The participant was required to respond to a target feature in each sub-task, being the word identity in the first sub-task but the font color in the second and third sub-tasks. Neighboring stimuli did not share the same target feature. The participant was given 45 s to name as many stimuli as possible in all three tasks. They should respond column-by-column (from top to bottom), and from left to right. If they managed to finish naming all six columns, they should resume from the top left corner until the time was up. Incorrect responses (e.g., a slip of tongue), if any, should be self-corrected immediately, in which case there would be no reduction in the score. Two measures, processing speed and inhibition, were derived based on the scores in the three sub-tasks (Fong et al., 2021).

S2.2 Digit span forward

A sequence comprising the digits 0-9 (in Cantonese) was presented at a rate of 2 s per digit (1 s per sound file, followed by one second of silence) via a loudspeaker in each trial. Participants were instructed to immediately recall the sequence in the same order. Three practice trials at a memory load of 3 were given, before the critical test began. Participants were given 3 trials for each load. The memory load of each sequence was increased successively from 4, until they were unable to correctly recall at least 2 out of 3 sequences. **Table S3** shows the complete list of digit sequences used. For load ≤ 10, each digit appeared at most once; for load > 10, each digit was repeated at most twice. The audio recording for the digits were selected from the CUSYL database (Lee et al., 2002), which contains the oral productions of all commonly used Cantonese syllables by four young speakers. The stimuli from a male speaker CS01M were used to construct the auditory stimuli. The single performance measure used was abbreviated as DigitSpan, the maximum load at which the participant was able to correctly respond to at least two out of three trials; a half mark was added if the participant were able to correctly respond to one out of the three trials at the next memory load (e.g., Cherry et al., 2012). This measure can be considered as an index of phonological STM (Fisk and Warr, 1996; Haarmann and Usher, 2001).

S2.3 Hong Kong List Learning Test

We adopted and computerized the randomized version of HKLLT (Chan and Kwok, 1998). In this test, the participant was asked to learn a memory list of sixteen disyllabic Cantonese words over three learning trials (Trials 1-3). These words belonged to four semantic categories (fruits, furniture, relatives, and countries). For each trial, the memory list would be presented at a speed of 1 word every 2 s via a loudspeaker; participants should freely recall the items immediately after list presentation. Participants were then unexpectedly asked to recall the word list at lags of 10 min (i.e., Trial 4) and 30 min (i.e., Trial 5). Finally, a recognition test would be given, in which participants should respond to "old" words by pressing the right button, and to "new" words by pressing the left button. There were 32 words in total (16 old; 16 new). Between Trial 3 and 4, participants were given a non-demanding interference task (one-back task). Similarly, between Trial 4 and 5, participants would play the game of Tower of Hanoi (Goel and Grafman,

Table S3. Digit sequences used in the digit span forward task. The memory load was varied from 4 (practice) to 14, and there were three trials (Trial 1-3) per load.

Load	Trial 1	Trial 2	Trial 3
4 (Practice)	7 1 8 6	2 6 5 4	7 0 8 5
5	8 3 1 6 5	9 8 6 5 1	7 4 6 0 8
6	8 2 7 0 4 1	7 6 0 1 8 2	7 9 4 0 3 2
7	8 9 3 6 5 2 4	2 8 7 3 0 9 6	4 0 6 1 7 3 9
8	2 3 7 5 4 0 1 8	5 8 6 9 3 0 7 1	3 8 6 5 1 0 4 9
9	6 9 8 1 7 2 4 3 0	0 7 2 8 5 3 9 6 1	4 2 5 1 0 9 3 6 7
10	4 3 6 7 0 1 2 5 9 8	6 9 8 3 2 1 4 5 0 7	0 4 3 1 7 2 9 8 5 6
11	2 7 3 1 5 9 4 8 6 0 2	4 8 7 6 1 5 3 6 2 0 9	2 5 0 7 1 3 4 8 6 9 0
12	0 8 5 7 3 4 6 2 5 0 9 1	4 1 9 3 7 1 6 2 6 5 0 8	3 2 6 8 4 9 7 8 0 4 5 1
13	3 1 2 7 0 5 3 8 7 9 4 6 2	7 1 7 9 6 3 9 5 2 8 4 5 0	5 2 9 7 4 3 8 9 6 4 1 5 0
14	4 5 8 3 7 8 6 1 9 5 4 2 6 0	0 9 5 1 9 3 7 4 3 1 2 6 2 8	3 5 6 4 3 9 1 6 9 8 5 2 7 0

1995), which involved the re-arrangement of a given stack of disks from one peg to another, under two rules: (a) only one disk could be re-arranged in each move, and (b) a smaller disk should always be placed above a larger one. The game began with three disks; a disk would be added each time the participant completed the rearrangement, until the time ran out. A real object version of Tower of Hanoi was used. One-back and Tower of Hanoi were used, because their non-verbal nature would minimize the interference with verbal episodic memory. The five free recall trials were analyzed as follows. For each recall trial, the individual response sequence was first manually transcribed; the responses in the sequence were then classified into correct responses, perseveration errors, and intrusion errors. Filler words (e.g., "umm") were not transcribed. The performance for each of the five trials (W1 to W5) was scored as the number of distinct correct responses. In the present study, the score at the fifth trial (i.e., the recall performance after a 30-minute lag) was used a single measure of verbal episodic memory.

S2.4 Picture naming

This test was developed in our previous work (Fong et al., 2020). All stimuli were line drawings chosen from the International Picture Naming Database; they have been formerly tested in a cross-linguistic norming study covering seven languages (Bates et al., 2003). Because Cantonese was not among the seven languages, a small-scale norming study was conducted to examine the naming latency and naming consistency of each item in an independent group of 27 young pilot participants aged 18-25. Only those stimuli that were disyllabic, with a z-score larger than -0.5 to 1.5 for naming latency, and a naming consistency of at least 75 % were kept, leaving 127 items out of 362 for further processing. Another group of 21 young pilot participants were rated to rate the goodness of depiction and familiarity of the words from a range of 1 to 5. Only stimuli with a z-score larger than -0.5 in both scales were kept, leaving a total of 89 stimuli for further selection. Only those with a z-score in visual complexity of between -1.5 to 1.5 were included in the test. Forty-two stimuli that matched all the criteria above were finally chosen. Each picture (7.5 cm $\times$ 7.5 cm) subtended a visual angle of about 6.0° on-screen, and was presented for 5 s. Participants were asked to name the picture as quickly and accurately as possible. Five practice trials were given. **Table S4** lists the stimulus properties of the stimuli chosen in this task, including RT (based on an independent group of younger participants), familiarity (based on a rating study), goodness of depiction (based on a rating study), visual complexity (as determined by file size), and log-frequency (based on Google page counts; accessed 2016-08-16).

Table S4. Basic information for the picture stimuli used in the picture naming task.

ID	English	Chinese	RT	Familiarity	Depiction	VC	LogFreq
1	Key	鎖匙	889.65	4.52	4.67	19769	6.34
2	Butterfly	蝴蝶	901.87	4.29	4.57	10948	8.11
3	Glasses	眼鏡	907.42	4.90	4.76	9671	8.20
4	Icecream cone	雪糕	918.91	4.67	4.38	6897	7.31
5	Balloon	汽球	921.94	4.67	4.71	6699	7.18
6	Finger	手指	941.87	4.81	4.43	7378	8.20
7	Toothbrush	牙刷	955.94	4.71	4.52	24154	7.54
8	Closet	衣櫃	962.93	4.86	4.76	6497	7.16
9	Corn	粟米	967.42	4.95	4.52	4794	6.53
10	Present	禮物	974.25	4.62	4.43	9564	7.84
11	Bicycle	單車	989.25	4.76	4.62	13179	7.50
12	Peanut	花生	1006.31	4.71	4.00	8638	7.79
13	Scissors	鉸剪	1055.57	4.76	4.67	10906	5.66
14	Mouse	老鼠	1062.24	4.81	4.90	19464	7.92
15	Ax	斧頭	1071.27	4.38	4.52	12268	6.31
16	Skateboard	滑板	1072.82	4.67	4.24	15399	7.45
17	Couch	梳化	1073.72	4.24	4.48	10326	6.33
18	Crown	皇冠	1079.65	4.14	4.67	11732	8.14
19	Clown	小丑	1090.75	4.76	4.76	17153	7.44
20	Tomato	蕃茄	1097.33	4.76	4.86	8495	7.01
21	Bandaid	膠布	1108.02	4.71	4.43	20904	6.27
22	Swing	韆鞦	1123.21	4.33	4.00	13738	7.58
23	Can	罐頭	1134.42	4.76	4.67	12976	6.90
24	Windmill	風車	1159.15	4.43	4.57	17113	7.86
25	Monkey	馬騮	1164.02	4.10	4.57	18958	5.63
26	Popcorn	爆谷	1225.57	4.67	4.10	11187	6.09
27	Alligator	鱷魚	1240.79	4.71	4.00	7209	7.08
28	Iron	熨斗	1255.94	4.29	4.48	6818	6.45
29	Shower	花洒	1321.49	4.29	4.33	16255	6.93
30	Sailboat	帆船	1328.41	4.10	4.24	12703	7.46
31	Bag	紙袋	1349.18	4.48	4.19	19378	7.44
32	Lipstick	唇膏	1354.82	4.24	4.24	6954	7.57
33	Cheese	芝士	1365.01	4.57	4.00	15933	7.35
34	Trophy	獎盃	1403.06	4.67	4.19	10764	7.21
35	Waiter	侍應	1411.68	4.71	4.14	13186	6.21
36	Branch	樹枝	1419.21	4.48	4.19	16228	6.99
37	Lobster	龍蝦	1428.28	4.62	4.19	14531	7.13
38	Crackers	餅乾	1470.02	4.57	4.52	21941	7.32
39	Yoyo	搖搖	1472.62	4.62	4.67	5179	6.52
40	Whale	鯨魚	1475.85	4.48	4.24	6267	6.74
41	Drill	電鑽	1504.37	4.10	4.19	13287	6.45
42	Scarf	頸巾	1519.18	4.19	4.38	15511	6.69

RT = reaction time, Depiction = goodness of depiction,
VC = visual complexity, LogFreq = log-Google frequency in base 10.

S2.5 Semantic fluency

In the semantic fluency task, sixteen categories were tested in separate questions. Each question began with the presentation of a crosshair sign for 1000 ms. After a blank of 500 ms, a description of a category (e.g., names of mammals) was displayed for 5000 ms, along with an example (e.g., tiger). Upon viewing the instruction “recording begins”, participants should verbally produce as many words as possible that fits the description of the category. The time limit was 60 s per question. A practice question (“means of transportation”) was given prior to the sixteen critical questions. All responses were recorded for detailed analysis. **Table S5** lists the category and example that were displayed on-screen. The order of the questions was randomized.

This test was developed in our previous work (Fong et al., 2020, 2021), and the same data transcription procedure was adopted. In brief, for each category, the complete list of unique responses from all participants was compiled. Intrusions (i.e., items not belonging to the semantic category tested) were identified by three independent judges, with disagreements resolved using the majority rule. To identify perseveration errors, unique responses that were considered synonymous by two of the three judges were counted as the same concept. For example, in Cantonese, the concept “monkey” can be referred to as either /maa5 lau1/ colloquially or /hau4 zi2/ in literary form. For each participant, the semantic fluency for each category was calculated as the number of correct concepts produced; the example given as part of the question, intrusion errors, and perseveration errors were not counted towards this score. The final score was calculated as the average semantic fluency across all sixteen categories.

Table S5. Semantic categories tested in the semantic fluency task. An example was given to illustrate the semantic category.

Semantic category	Example
Names of mammals	Tiger
Names of non-mammals	Ant
Names of fruits	Apple
Names of kitchenware	Chopsticks
Names of tools	Hammer
Names of stationery	Pencil
Names of electrical appliances	Television
Names of toys	Yoyo
Names of countries	China
Names of subway stations	Hung Hom
Personal particulars often required for form-filling	Name
Names of occupations common in Hong Kong	Doctor
Units of time	Week
Elements for success	Luck
Matter and things related to finance management	Account
Matter and things related to learning and studying	Grade

S2.6 Raven’s standard progressive matrices

Raven’s Standard Progressive Matrices comprised five sections of twelve questions each, arranged in an increasing level of difficulty (Raven and Court, 1998). Due to time limitation and the need to prevent repetition, the original test was divided into two versions by selecting only either the odd- or even- numbered questions. Participants were given 22.5 min (half the time allocated to the full test) and a sheet of paper to record their answer. The percentage of questions answered correctly was obtained; the resultant measure, RavenAcc, was defined as the standardized score of accuracy, computed separately for the odd-version and

even-version (Fong et al., 2020). The scores were standardized based on the norm from an independent group of 57 participants aged 60-70 (odd: $M = 23.07$, $SD = 2.89$, even: $M = 21.30$, $SD = 4.39$).

S3 PHONOLOGICAL TEST BATTERY

Two phonological tests (discrimination and spoonerism) each were run for all three languages—Cantonese (L1), English (L2), and Italian (L3). Each test began with two practice trials, for which correct answers were given as the feedback in case of wrong answers. For the Cantonese tests, the stimuli were spoken syllables produced by the speaker "CS04F" in the CUSYL database (Lee et al., 2002). The auditory stimuli for English and Italian were downloaded from the ttsmp3 webpage, https://ttsmp3.com/text-to-speech/Italian/. For English, the British English voice "Brian" was chosen to synthesize the stimuli, while for Italian, the voice "Giorgio" was used. For Cantonese and English, but stimuli were all real words. In contrast, for Italian, nonwords were used to make sure that any changes in the phonological performance of Italian would not arise simply due to a stimulus overlap with the study materials.

S3.1 Phoneme discrimination

For the discrimination task, regardless of language, participants heard four auditory stimuli in succession in each trial (*c.f.* Koda, 1998). Three of the stimuli shared the same segmental feature or suprasegmental feature (in the case of Cantonese tone), with the fourth not sharing the feature in question. The number of blocks and trials for each language examined was different, due to the different number of distinctive segmental and suprasegmental feature in each language. The Cantonese discrimination task was divided into three blocks, in which the feature in question was initial consonant (25 trials), vowel (12 trials), and tone (6 trials), respectively. The English discrimination task was divided into two blocks, in which the feature in question was initial consonant (26 trials) and vowel (12 trials). The Italian discrimination task was divided into two blocks, in which the feature in question was also initial consonant (26 trials) and vowel (12 trials). All stimuli tested were monosyllabic. See **Tables S6, S7, S8** for the complete set of stimuli for each language version.

S3.2 Spoonerism

For spoonerism, participants listened to a pair of words in succession. Their task was to exchange the initial consonant of the two words. **Table S9** provides the list of questions for each language version. There were a maximum of 15 trials in all three versions. Each correctly produced word was worth 1 point, so that the maximum score in each version was 30 points. However, the test would be terminated if the participants failed to score any point in three consecutive trials.

Table S6. Cantonese discrimination task. All stimuli were transcribed in Jyutping, a phonetic transcription system of Cantonese endorsed by the Linguistic Society of Hong Kong. The task was to judge, among the four choices (Choice 1-4), the one that differs from the remaining in terms of the initial consonant / vowel / tone.

Feature	Choice 1	Choice 2	Choice 3	Choice 4
Consonant	faai3	saai3	saai2	seoi3
	gaa1	gaa3	gwaa1	gu1
	gei3	koi3	kei3	kei5
	hung4	fong4	fung6	fung4
	wai1	wui5	lai5	wai5
	lin6	lyun2	lyun6	jyun6
	baau2	paau3	pou2	paau2
	kwaang3	kwong3	kong3	kwong4
	nau5	lou5	nou5	nou6
	mei6	mei5	nei5	mui5
	hiu2	hou2	hiu1	siu2
	dang1	dang2	deng1	zang1
	ban2	gan2	bun2	ban3
	paau4	piu4	piu1	kiu4
	do1	to5	to1	taa1
	pou4	tou4	tau4	tou5
	geoi3	deoi2	geoi2	goi2
	keoi5	kei4	teoi4	keoi4
	ngau4	mau5	ngaau5	ngau5
	gwai1	kwai1	gwai6	gwaai1
	cin4	can1	cin1	tin1
	zi3	ce3	ze6	ze3
	joek6	jik1	wik6	jik6
	doi6	daai6	noi6	doi2
	naan4	naan6	nyun6	ngaan6
Vowel	loeng4	koeng4	long4	loeng6
	ce4	ci3	ci4	ji4
	gyun6	gun3	gyun3	syun3
	wu4	wu1	fu1	waa1
	fo3	fo2	gwo3	fu3
	bin2	waan2	baan2	baan1
	mun5	min6	bun6	mun6
	long5	leng5	teng5	leng3
	zi6	ji5	jyu6	ji6
	geng2	geng1	goeng1	deng1
Tone	keoi1	deoi3	deoi1	dai1
	lau5	lou2	lou5	pou5
	mui6	wui6	maai6	mui4
	sin6	bin3	saan3	sin3
	jyun4	can4	jan4	jan1
	fu2	fo2	fu5	gu2

Table S7. English discrimination task. All stimuli chosen are real monosyllabic words. The task was to judge, among the four choices (Choice 1-4), the one that differs from the remaining in terms of the initial consonant / vowel.

Feature	Choice 1	Choice 2	Choice 3	Choice 4
Consonant	reap	leap	lead	lap
	gut	cup	cut	cart
	bus	buck	duck	book
	theme	sum	seek	seem
	fouled	fold	hold	foam
	gay	gate	day	guy
	let	yacht	yes	yet
	chip	gin	chin	churn
	dumb	ditch	dutch	touch
	park	put	tart	part
	shot	sheet	sheep	cheat
	wall	yell	wet	well
	hall	call	hell	horn
	zip	sit	sip	soup
	rate	rake	right	wait
	sham	gym	jam	jazz
	them	zen	than	then
	cool	tooth	tall	tool
	pay	bay	buy	bake
	vain	vice	vine	thine
	knock	mock	nod	neck
	shark	sock	shock	shop
	thick	thin	thorn	fin
	moot	meet	mood	boot
	fan	fun	van	fat
	lane	life	nine	line
Vowel	tall	tooth	tool	cool
	hurt	bird	hard	herd
	sad	math	mad	mud
	feel	feet	fit	cheat
	port	cot	court	call
	dead	dad	deck	red
	sit	mill	sill	sell
	shark	sheep	harp	sharp
	wood	food	word	whoop
	duck	luck	love	look
	get	knot	got	golf
	pool	pull	push	wool

Table S8. Italian discrimination task. All stimuli were transcribed using the International Phonetic Alphabet (IPA). The task was to judge, among the four choices (Choice 1-4), the one that differs from the remaining in terms of the initial consonant / vowel.

Feature	Choice 1	Choice 2	Choice 3	Choice 4
Consonant	man	nan	nal	nun
	ba	bil	bal	pal
	ɲan	ʎan	ɲa	ɲon
	tel	kil	kel	ken
	ʃel	ʃul	sel	ʃe
	tsɔn	tsɔl	tsan	tɔn
	ʎɔl	ʎel	ʎɔn	lɔl
	tul	til	dil	tin
	tʃen	tʃun	tʃul	ʃun
	dzal	dzɔ	dzɔl	dɔl
	vel	fel	fal	fen
	vu	bu	vɔ	vun
	gɔn	kɔ	dʒi	gɔ
	ʃin	ʃial	fil	ʃil
	tsi	dze	tsen	tse
	mul	mel	mu	ɲul
	dʒan	tʃal	dʒal	dʒɔl
	pun	pɔl	fɔn	pɔn
	dʒil	dzin	dzel	dzil
	dul	dun	dan	gun
	tsa	tʃu	tʃan	tʃa
	sil	sin	tsin	sen
	nen	rel	ren	rɔn
	nil	nin	ɲil	nɔl
	lil	rul	lul	lun
Vowel	tsa	tsu	mu	tsul
	tel	tɔl	ten	rel
	ʃe	ʎe	ʃel	ʃu
	sil	dʒil	sal	sin
	fɔ	ɲɔn	ɲɔ	ɲi
	dan	len	lal	lan
	nul	kul	kil	ku
	ban	ba	bɔ	tʃa
	pen	dzin	pi	pin
	dʒo	vɔ	vɔl	vu

Table S9. The Spoonerism task in all three language versions (Cantonese, English, and Italian). The task was to swap the initial consonants of the two words (Cantonese and English) or non-words (Italian).

Cantonese		English		Italian	
pei3	kai1	peak	west	vu	bɔ
cin4	lau4	share	dark	ʃu	pen
fan2	bui3	fast	pool	tʃu	na
luk6	miu5	net	vow	dʒi	fɔ
bo3	gwaa2	thumb	sink	tsil	ru
mou2	dei6	mouse	hall	dzil	ka
zoi6	ngung1	bay	there	fe	ɲil
je4	ci5	white	birth	mel	tɔn
wui5	paai4	yank	rear	den	lul
syun3	haau2	lip	zone	ʎal	sen
haai5	nou6	ghost	tame	pa	dʒi
keoi5	se2	tour	look	ɲa	ʎul
kwaa1	fong4	rude	mug	kɔ	tsi
giu1	teoi3	cog	joy	rɔl	dan
dau1	zat6	cheer	fat	gɔn	ʃe

S4 ANATOMICAL MEASURES

Table S10 summarizes, for each cortical ROI, the four anatomical measures extracted (thickness, area, volume, and curvature). **Table S11** summarizes the normalized volumes of all sub-cortical ROIs.

S5 LINEAR MIXED-EFFECTS MODELLING

Table S12 summarizes the results of the likelihood ratio tests for comparing the cortical models of the in-class Ita-to-Can performance against the baseline demographic model. Four of the models were marginally significant (p <0.10, FDR-corrected), including the models based on (1) left pars orbitalis, (2) left caudal middle frontal cortex, (3) right insula, and (4) left entorhinal cortex. Type III sums of squares analyses were conducted as the post hoc follow-up tests (**Table S13**). Regarding (1) left pars orbitalis, the performance was positively associated with adjusted volume, t = 3.70, p = 0.001, and adjusted curvature, t = 3.35, p = 0.003. The interaction of Week $\times$ adjusted volume was significant; *post hoc* trend analysis showed that the association was significant in all five weeks, df = 26.7, ts $>$ 2.74, ps $<$ 0.011, and that the difference in slope was significant between week 2 and week 3, t = $-$2.91, p = 0.037. Regarding (2) right insula, the performance was significantly associated with adjusted area, t = $-$5.95, p $<$ 0.001, and adjusted volume, t = 2.79, p = 0.011. No interaction effect was significant. Regarding (3) left caudal middle frontal cortex, the performance was significantly associated with adjusted volume, t = 3.56, p = 0.002, and adjusted curvature, t = 3.51, p = 0.002. Week $\times$ adjusted area was significant. *Post hoc* trend analysis showed that although the association was non-significant in any week, i.e., the slope was not significantly different from zero. However, the slope was significantly more steep in Week 2 and Week 5, compared to that in Week 1. Regarding (4) left entorhinal cortex, the performance was significantly associated with adjusted curvature, t = $-$4.05, p $<$ 0.001, which in turn interacted with week; *post hoc* trend analysis showed that the association was significant in all five weeks, but the difference in association was non-significant across all pairs of weeks, ps $>$ 0.10.

Similarly, **Tables S14, S16** summarize the likelihood ratio tests for the in-class Can-to-Ita and final test performance. **Tables S15, S17** show the Type III sums of squares analyses conducted in each of the significant / marginally significant models.

Table S10. Summary statistics of the four cortical measures, measured at different cortical ROIs.

Cortical region	Thickness (mm)				Area (mm^2)				Volume (mm^3)				Curvature			
	Mean	*SD*	*Min*	*Max*	*Mean*	*SD*	*Min*	*Max*	*Mean*	*SD*	*Min*	*Max*	*Mean*	*SD*	*Min*	*Max*
L bankssts	2.2	0.1	2	2.4	912.9	156.2	669	1147	1940.4	384.3	1431	2965	0.092	0.013	0.069	0.117
L caudalanteriorcingulate	2.2	0.2	1.8	2.6	562	116	372	763	1339.2	317.5	921	1870	0.118	0.017	0.082	0.14
L caudalmiddlefrontal	2.3	0.1	2.2	2.5	2080.1	329.2	1677	3125	5267.6	803.2	4153	7502	0.109	0.006	0.1	0.121
L entorhinal	3.1	0.2	2.8	3.5	396.7	69	242	583	1755.2	285.7	1033	2294	0.116	0.01	0.098	0.133
L fusiform	2.5	0.1	2.4	2.7	2879.3	233.6	2465	3296	8200.4	788.1	6571	9640	0.125	0.008	0.108	0.145
L middletemporal	2.5	0.1	2.3	2.7	3162.6	512.3	2485	4128	9842	1548.8	7774	12486	0.122	0.008	0.105	0.136
L parahippocampal	2.3	0.2	2	2.9	634.2	106.4	518	1011	1735	249.6	1398	2620	0.085	0.01	0.067	0.105
L parsopercularis	2.3	0.1	2.1	2.4	1425.7	189.8	1072	1731	3654.8	538	2704	4573	0.113	0.01	0.093	0.128
L parsorbitalis	2.5	0.1	2.3	2.6	622.5	74.9	461	756	1926.2	246.1	1397	2381	0.139	0.012	0.114	0.16
L parstriangularis	2.3	0.1	2.1	2.4	1253.2	203.4	803	1566	3179.9	547.3	2149	4347	0.122	0.009	0.099	0.135
L precentral	2.4	0.1	2.2	2.6	4652.8	374.7	3936	5520	12163.2	970.2	10168	13882	0.101	0.006	0.092	0.118
L rostralanteriorcingulate	2.4	0.2	2.1	2.9	756.5	169.7	358	1015	2048.9	488.6	1110	2934	0.127	0.017	0.096	0.156
L rostralmiddlefrontal	2.2	0.1	2	2.3	5455	595.8	4158	6602	13118.5	1532.1	10514	16373	0.134	0.009	0.119	0.147
L supramarginal	2.3	0.1	2.1	2.4	3744.8	587.3	2486	4986	9390.6	1404.2	6390	12137	0.122	0.006	0.112	0.136
L temporalpole	3.3	0.2	2.9	3.8	540.5	41.1	465	644	2502.2	314.9	2112	3503	0.141	0.017	0.121	0.171
L insula	2.7	0.1	2.4	3.1	2319.2	223.8	1934	2872	6307	543.2	5361	7232	0.123	0.01	0.105	0.157
R bankssts	2.3	0.1	2	2.5	810.1	116.4	564	1037	1753.2	241	1287	2253	0.096	0.01	0.069	0.112
R caudalanteriorcingulate	2.2	0.2	1.8	2.6	658	101.2	483	875	1620.1	253.2	1202	2165	0.127	0.013	0.111	0.154
R caudalmiddlefrontal	2.4	0.1	2.2	2.6	2022.8	226.4	1681	2305	5272.5	602.4	4342	6408	0.11	0.007	0.097	0.121
R entorhinal	3.2	0.2	3	3.6	371.4	54.8	302	472	1759.4	219.8	1334	2228	0.128	0.013	0.108	0.163
R fusiform	2.5	0.1	2.3	2.7	2813.3	380	2123	3731	8168.8	1330.2	5875	11533	0.125	0.009	0.101	0.142
R middletemporal	2.6	0.1	2.4	2.8	3349.8	456	2466	4097	10535.5	1310.5	8110	12656	0.12	0.008	0.107	0.132
R parahippocampal	2.3	0.2	2.1	2.7	589.2	61.4	466	683	1578.3	199.4	1276	2032	0.087	0.007	0.077	0.099
R parsopercularis	2.3	0.1	2.2	2.5	1253.9	141	1020	1501	3192.4	378.4	2669	4009	0.112	0.007	0.098	0.122
R parsorbitalis	2.4	0.1	2.2	2.8	787	90.5	598	919	2334.9	304	1751	2819	0.147	0.011	0.125	0.163
R parstriangularis	2.2	0.1	2	2.4	1511	216.8	1155	1829	3731.3	509.3	3029	4577	0.123	0.009	0.108	0.142
R precentral	2.4	0.1	2.2	2.7	4642.2	352.2	3998	5219	12020.7	928.5	10678	13644	0.1	0.005	0.091	0.111
R rostralanteriorcingulate	2.5	0.2	2.3	2.8	548.7	131.9	325	826	1606.8	383.9	841	2383	0.131	0.011	0.112	0.15
R rostralmiddlefrontal	2.2	0.1	2.1	2.4	5569	735.9	4236	7258	13252	1711.2	10034	16721	0.135	0.008	0.123	0.153
R supramarginal	2.3	0.1	2.1	2.5	3346.3	489.2	2534	4164	8355.2	1132.4	5748	10129	0.118	0.006	0.108	0.127
R temporalpole	3.4	0.3	2.9	4	532.2	60.5	433	692	2580.1	463.5	1888	3617	0.146	0.013	0.12	0.174
R insula	2.7	0.1	2.4	3	2215.4	240.1	1770	2660	6087	568.1	4882	7014	0.12	0.014	0.107	0.174

Table S11. Summary statistics of normalized sub-cortical volumes, measured at different sub-cortical ROIs.

Sub-cortical region	*Mean*	*SD*	*Min*	*Max*
L thalamus	0.44	0.04	0.36	0.5
L caudate	0.22	0.02	0.16	0.27
L putamen	0.30	0.04	0.18	0.35
L hippocampus	0.24	0.02	0.18	0.28
R thalamus	0.42	0.03	0.35	0.48
R caudate	0.22	0.02	0.19	0.28
R putamen	0.31	0.04	0.19	0.35
R hippocampus	0.25	0.01	0.22	0.27

Table S12. Model comparisons between the cortical models of Ita-to-Can performance and the corresponding baseline demographic model.

Region	*AIC*	*BIC*	*logLik*	*r2m*	*r2c*	χ^2	*df*	*p (unc)*	*p (FDR)*	
baseline	746.73	772.79	-363.37	0.22	0.91					
L rostralmiddlefrontal	763.84	841.99	-351.92	0.54	0.93	22.90	20	0.294	0.495	ns
L caudalmiddlefrontal	747.91	826.07	-343.96	0.63	0.94	38.82	20	0.007	0.075	†
L parsopercularis	771.61	849.77	-355.81	0.32	0.93	15.12	20	0.769	0.821	ns
L parstriangularis	751.90	830.06	-345.95	0.59	0.93	34.83	20	0.021	0.112	ns
L parsorbitalis	742.99	821.14	-341.49	0.67	0.94	43.75	20	0.002	0.052	†
L insula	771.17	849.33	-355.59	0.43	0.92	15.56	20	0.743	0.821	ns
L temporalpole	759.19	837.35	-349.60	0.53	0.93	27.54	20	0.121	0.276	ns
L bankssts	756.29	834.44	-348.14	0.43	0.94	30.45	20	0.063	0.201	ns
L supramarginal	763.13	841.29	-351.57	0.58	0.92	23.60	20	0.260	0.468	ns
L entorhinal	749.27	827.42	-344.63	0.58	0.94	37.46	20	0.010	0.082	†
L parahippocampal	768.51	846.66	-354.25	0.32	0.93	18.23	20	0.573	0.733	ns
L fusiform	773.93	852.08	-356.96	0.29	0.93	12.81	20	0.885	0.885	ns
L rostralanteriorcingulate	763.19	841.35	-351.60	0.48	0.93	23.54	20	0.263	0.468	ns
L caudalanteriorcingulate	769.73	847.89	-354.87	0.39	0.93	17.00	20	0.653	0.774	ns
L precentral	766.58	844.74	-353.29	0.48	0.93	20.15	20	0.448	0.630	ns
L middletemporal	754.23	832.38	-347.11	0.49	0.94	32.51	20	0.038	0.163	ns
R rostralmiddlefrontal	754.50	832.65	-347.25	0.53	0.93	32.23	20	0.041	0.163	ns
R caudalmiddlefrontal	763.01	841.16	-351.50	0.34	0.93	23.73	20	0.255	0.468	ns
R parsopercularis	771.22	849.38	-355.61	0.30	0.93	15.51	20	0.746	0.821	ns
R parstriangularis	762.77	840.92	-351.38	0.33	0.93	23.97	20	0.244	0.468	ns
R parsorbitalis	751.16	829.31	-345.58	0.33	0.94	35.58	20	0.017	0.110	ns
R insula	747.47	825.63	-343.74	0.75	0.93	39.26	20	0.006	0.075	†
R temporalpole	766.51	844.67	-353.26	0.29	0.93	20.22	20	0.444	0.630	ns
R bankssts	758.83	836.99	-349.42	0.44	0.93	27.90	20	0.112	0.276	ns
R supramarginal	764.48	842.63	-352.24	0.35	0.93	22.26	20	0.327	0.523	ns
R entorhinal	755.31	833.46	-347.65	0.42	0.94	31.43	20	0.050	0.177	ns
R parahippocampal	759.00	837.16	-349.50	0.45	0.93	27.73	20	0.116	0.276	ns
R fusiform	758.91	837.06	-349.45	0.46	0.93	27.83	20	0.114	0.276	ns
R rostralanteriorcingulate	769.04	847.19	-354.52	0.39	0.93	17.70	20	0.607	0.748	ns
R caudalanteriorcingulate	768.08	846.23	-354.04	0.35	0.93	18.66	20	0.544	0.726	ns
R precentral	773.07	851.23	-356.54	0.26	0.93	13.66	20	0.847	0.875	ns
R middletemporal	766.66	844.81	-353.33	0.32	0.93	20.08	20	0.453	0.630	ns

***, p <0.001, **, p <0.01, *, p <0.05, †, marginal, ns, non-significant.

Table S13. Type III sums of squares analyses on the marginally significant cortical Ita-to-Can models.

**Term**	***Sum Sq***	***Mean Sq***	***NumDF***	***DenDF***	***F***	***p***	
L parsorbitalis							
Week	3175.31	793.83	4	80.0	27.37	< 0.001	***
Age	13.89	13.89	1	20.0	0.48	0.497	ns
Education	40.39	40.39	1	20.0	1.39	0.252	ns
Gender	1.80	1.80	1	20.0	0.06	0.806	ns
thickness	2.71	2.71	1	20.0	0.09	0.763	ns
area	74.29	74.29	1	20.0	2.56	0.125	ns
volume	396.33	396.33	1	20.0	13.67	0.001	**
meancurv	326.27	326.27	1	20.0	11.25	0.003	**
Week:thickness	142.25	35.56	4	80.0	1.23	0.306	ns
Week:area	224.40	56.10	4	80.0	1.93	0.113	ns
Week:volume	297.97	74.49	4	80.0	2.57	0.044	*
Week:meancurv	199.28	49.82	4	80.0	1.72	0.154	ns
L caudalmiddlefrontal							
Week	3139.81	784.95	4	80.0	26.22	< 0.001	***
Age	23.42	23.42	1	20.0	0.78	0.387	ns
Education	8.12	8.12	1	20.0	0.27	0.608	ns
Gender	148.58	148.58	1	20.0	4.96	0.038	*
thickness	6.48	6.48	1	20.0	0.22	0.647	ns
area	3.93	3.93	1	20.0	0.13	0.721	ns
volume	380.02	380.02	1	20.0	12.70	0.002	**
meancurv	369.62	369.62	1	20.0	12.35	0.002	**
Week:thickness	82.53	20.63	4	80.0	0.69	0.601	ns
Week:area	511.79	127.95	4	80.0	4.27	0.003	**
Week:volume	170.07	42.52	4	80.0	1.42	0.235	ns
Week:meancurv	24.76	6.19	4	80.0	.21	0.934	ns
R insula							
Week	3100.77	775.19	4	80.0	23.03	< 0.001	***
Age	361.79	361.79	1	20.0	10.75	0.004	**
Education	25.38	25.38	1	20.0	0.75	0.395	ns
Gender	69.39	69.39	1	20.0	2.06	0.167	ns
thickness	13.43	13.43	1	20.0	0.40	0.535	ns
area	1192.01	1192.01	1	20.0	35.41	< 0.001	***
volume	262.57	262.57	1	20.0	7.80	0.011	*
meancurv	52.65	52.65	1	20.0	1.56	0.226	ns
Week:thickness	183.77	45.94	4	80.0	1.36	0.254	ns
Week:area	149.61	37.40	4	80.0	1.11	0.357	ns
Week:volume	45.17	11.29	4	80.0	0.34	0.853	ns
Week:meancurv	112.45	28.11	4	80.0	0.84	0.507	ns
L entorhinal							
Week	3263.43	815.86	4	80.0	27.72	< 0.001	***
Age	1.38	1.38	1	20.0	0.05	0.831	ns
Education	169.87	169.87	1	20.0	5.77	0.026	*
Gender	129.48	129.48	1	20.0	4.40	0.049	*
thickness	51.07	51.07	1	20.0	1.73	0.203	ns
area	15.39	15.39	1	20.0	0.52	0.478	ns
volume	39.02	39.02	1	20.0	1.33	0.263	ns
meancurv	483.57	483.57	1	20.0	16.43	0.001	***
Week:thickness	170.92	42.73	4	80.0	1.45	0.225	ns
Week:area	164.05	41.01	4	80.0	1.39	0.244	ns
Week:volume	200.31	50.08	4	80.0	1.70	0.158	ns
Week:meancurv	293.78	73.44	4	80.0	2.50	0.049	*

***, p <0.001, **, p <0.01, *, p <0.05, †, marginal, ns, non-significant.

Table S14. Cortical models for Can-to-Ita performance.

Region	*AIC*	*BIC*	*logLik*	*r2m*	*r2c*	χ^2	*df*	*p (unc)*	*p (FDR)*	
baseline	779.00	805.05	-379.50	0.25	0.90					
L rostralmiddlefrontal	796.01	874.17	-368.01	0.36	0.92	22.99	20	0.289	0.530	ns
L caudalmiddlefrontal	779.93	858.08	-359.96	0.56	0.93	39.08	20	0.007	0.035	*
L parsopercularis	802.38	880.54	-371.19	0.36	0.92	16.62	20	0.677	0.747	ns
L parstriangularis	782.35	860.51	-361.18	0.47	0.93	36.65	20	0.013	0.052	†
L parsorbitalis	739.46	817.62	-339.73	0.60	0.96	79.54	20	< 0.001	< 0.001	***
L insula	791.36	869.52	-365.68	0.49	0.92	27.64	20	0.118	0.262	ns
L temporalpole	782.89	861.04	-361.44	0.64	0.92	36.11	20	0.015	0.053	†
L bankssts	795.10	873.25	-367.55	0.46	0.92	23.91	20	0.246	0.493	ns
L supramarginal	801.96	880.12	-370.98	0.43	0.91	17.04	20	0.650	0.743	ns
L entorhinal	778.46	856.61	-359.23	0.70	0.92	40.55	20	0.004	0.027	*
L parahippocampal	798.79	876.95	-369.40	0.39	0.92	20.21	20	0.445	0.678	ns
L fusiform	801.39	879.54	-370.69	0.30	0.92	17.62	20	0.613	0.726	ns
L rostralanteriorcingulate	796.34	874.49	-368.17	0.44	0.92	22.67	20	0.305	0.530	ns
L caudalanteriorcingulate	799.88	878.03	-369.94	0.36	0.92	19.12	20	0.514	0.684	ns
L precentral	790.46	868.62	-365.23	0.39	0.93	28.54	20	0.097	0.239	ns
L middletemporal	799.39	877.55	-369.70	0.42	0.92	19.61	20	0.483	0.684	ns
R rostralmiddlefrontal	798.54	876.69	-369.27	0.43	0.92	20.46	20	0.429	0.678	ns
R caudalmiddlefrontal	803.11	881.27	-371.56	0.36	0.92	15.89	20	0.723	0.772	ns
R parsopercularis	796.52	874.68	-368.26	0.42	0.92	22.48	20	0.315	0.530	ns
R parstriangularis	784.01	862.16	-362.00	0.30	0.94	34.99	20	0.020	0.064	†
R parsorbitalis	773.79	851.94	-356.89	0.36	0.94	45.22	20	0.001	0.016	*
R insula	775.95	854.10	-357.97	0.72	0.92	43.05	20	0.002	0.019	*
R temporalpole	800.28	878.43	-370.14	0.27	0.92	18.73	20	0.540	0.684	ns
R bankssts	785.97	864.12	-362.98	0.41	0.93	33.04	20	0.033	0.089	ns
R supramarginal	810.16	888.32	-375.08	0.31	0.91	8.84	20	0.985	0.985	ns
R entorhinal	785.88	864.03	-362.94	0.49	0.93	33.13	20	0.033	0.089	†
R parahippocampal	800.21	878.37	-370.11	0.44	0.92	18.79	20	0.535	0.684	ns
R fusiform	800.53	878.68	-370.26	0.40	0.92	18.48	20	0.556	0.684	ns
R rostralanteriorcingulate	791.54	869.70	-365.77	0.39	0.93	27.46	20	0.123	0.262	ns
R caudalanteriorcingulate	776.55	854.71	-358.28	0.32	0.94	42.45	20	0.002	0.019	*
R precentral	803.51	881.67	-371.76	0.33	0.92	15.49	20	0.748	0.772	ns
R middletemporal	781.39	859.55	-360.70	0.33	0.94	37.61	20	0.010	0.045	*

***, p <0.001, **, p <0.01, *, p <0.05, †, marginal, ns, non-significant.

Table S15. Type III sums of squares analyses conducted on the significant cortical Can-to-Ita models.

Term	*Sum Sq*	*Mean Sq*	*NumDF*	*DenDF*	*F*	*p*	
L parsorbitalis							
Week	4841.82	1210.46	4	80.0	49.19	< 0.001	***
Age	21.64	21.64	1	20.0	0.88	0.360	ns
Education	39.11	39.11	1	20.0	1.59	0.222	ns
Gender	7.65	7.65	1	20.0	0.31	0.583	ns
thickness	4.54	4.54	1	20.0	0.18	0.672	ns
area	135.97	135.97	1	20.0	5.53	0.029	*
volume	6.98	6.98	1	20.0	0.28	0.600	ns
meancurv	225.77	225.77	1	20.0	9.17	0.007	**
Week:thickness	214.91	53.73	4	80.0	2.18	0.078	†

Week:area	396.71	99.18	4	80.0	4.03	0.005	**
Week:volume	1831.44	457.86	4	80.0	18.61	< 0.001	***
Week:meancurv	168.08	42.02	4	80.0	1.71	0.156	ns
R parsorbitalis							
Week	4718.35	1179.59	4	80.0	35.14	< 0.001	***
Age	86.83	86.83	1	20.0	2.59	0.123	ns
Education	0.02	0.02	1	20.0	0.00	0.983	ns
Gender	24.90	24.90	1	20.0	0.74	0.399	ns
thickness	7.59	7.59	1	20.0	0.23	0.639	ns
area	17.44	17.44	1	20.0	0.52	0.479	ns
volume	0.04	0.04	1	20.0	0.00	0.973	ns
meancurv	70.86	70.86	1	20.0	2.11	0.162	ns
Week:thickness	47.69	11.92	4	80.0	0.36	0.840	ns
Week:area	46.37	11.59	4	80.0	0.35	0.846	ns
Week:volume	1753.97	438.49	4	80.0	13.06	< 0.001	***
Week:meancurv	46.48	11.62	4	80.0	0.35	0.846	ns
R insula							
Week	4686.06	1171.52	4	80.0	26.40	< 0.001	***
Age	438.65	438.65	1	20.0	9.88	0.005	**
Education	90.13	90.13	1	20.0	2.03	0.170	ns
Gender	212.72	212.72	1	20.0	4.79	0.041	*
thickness	40.62	40.62	1	20.0	0.92	0.350	ns
area	693.14	693.14	1	20.0	15.62	0.001	***
volume	744.22	744.22	1	20.0	16.77	0.001	***
meancurv	296.00	296.00	1	20.0	6.67	0.018	*
Week:thickness	260.94	65.24	4	80.0	1.47	0.219	ns
Week:area	122.30	30.57	4	80.0	0.69	0.602	ns
Week:volume	600.40	150.10	4	80.0	3.38	0.013	*
Week:meancurv	45.99	11.50	4	80.0	0.26	0.903	ns
R caudalanteriorcingulate							
Week	4681.63	1170.41	4	80.0	34.24	< 0.001	***
Age	14.88	14.88	1	20.0	0.44	0.517	ns
Education	24.33	24.33	1	20.0	0.71	0.409	ns
Gender	6.24	6.24	1	20.0	0.18	0.674	ns
thickness	0.71	0.71	1	20.0	0.02	0.887	ns
area	27.75	27.75	1	20.0	0.81	0.378	ns
volume	12.07	12.07	1	20.0	0.35	0.559	ns
meancurv	0.37	0.37	1	20.0	0.01	0.918	ns
Week:thickness	88.80	22.20	4	80.0	0.65	0.629	ns
Week:area	961.83	240.46	4	80.0	7.03	< 0.001	***
Week:volume	338.93	84.73	4	80.0	2.48	0.051	†
Week:meancurv	455.34	113.83	4	80.0	3.33	0.014	*
L entorhinal							
Week	4496.30	1124.08	4	80.0	25.12	< 0.001	***
Age	1.20	1.20	1	20.0	0.03	0.871	ns
Education	399.42	399.42	1	20.0	8.92	0.007	**
Gender	242.40	242.40	1	20.0	5.42	0.031	*
thickness	587.03	587.03	1	20.0	13.12	0.002	**

area	7.53	7.53	1	20.0	0.17	0.686	ns
volume	7.91	7.91	1	20.0	0.18	0.679	ns
meancurv	1191.48	1191.48	1	20.0	26.62	< 0.001	***
Week:thickness	197.59	49.40	4	80.0	1.10	0.361	ns
Week:area	183.88	45.97	4	80.0	1.03	0.398	ns
Week:volume	89.17	22.29	4	80.0	0.50	0.737	ns
Week:meancurv	528.78	132.19	4	80.0	2.95	0.025	*
L caudalmiddlefrontal							
Week	4714.65	1178.66	4	80.0	29.17	< 0.001	***
Age	27.71	27.71	1	20.0	0.69	0.417	ns
Education	27.84	27.84	1	20.0	0.69	0.416	ns
Gender	36.49	36.49	1	20.0	0.90	0.353	ns
thickness	248.12	248.12	1	20.0	6.14	0.022	*
area	18.55	18.55	1	20.0	0.46	0.506	ns
volume	12.12	12.12	1	20.0	0.30	0.590	ns
meancurv	304.44	304.44	1	20.0	7.53	0.012	*
Week:thickness	1118.41	279.60	4	80.0	6.92	< 0.001	***
Week:area	92.78	23.19	4	80.0	0.57	0.682	ns
Week:volume	109.51	27.38	4	80.0	0.68	0.609	ns
Week:meancurv	26.66	6.66	4	80.0	0.16	0.956	ns
R middletemporal							
Week	4685.46	1171.37	4	80.0	32.15	< 0.001	***
Age	12.24	12.24	1	20.0	0.34	0.569	ns
Education	26.09	26.09	1	20.0	0.72	0.407	ns
Gender	6.56	6.56	1	20.0	0.18	0.676	ns
thickness	33.53	33.53	1	20.0	0.92	0.349	ns
area	13.11	13.11	1	20.0	0.36	0.555	ns
volume	7.68	7.68	1	20.0	0.21	0.651	ns
meancurv	0.74	0.74	1	20.0	0.02	0.888	ns
Week:thickness	264.91	66.23	4	80.0	1.82	0.134	ns
Week:area	491.06	122.77	4	80.0	3.37	0.013	*
Week:volume	651.18	162.79	4	80.0	4.47	0.003	**
Week:meancurv	257.76	64.44	4	80.0	1.77	0.143	ns
L triangularis							
Week	4924.94	1231.23	4	80.0	31.26	< 0.001	***
Age	74.23	74.23	1	20.0	1.89	0.185	ns
Education	26.09	26.09	1	20.0	0.66	0.425	ns
Gender	36.20	36.20	1	20.0	0.92	0.349	ns
thickness	31.06	31.06	1	20.0	0.79	0.385	ns
area	100.08	100.08	1	20.0	2.54	0.127	ns
volume	173.65	173.65	1	20.0	4.41	0.049	*
meancurv	10.52	10.52	1	20.0	0.27	0.611	ns
Week:thickness	166.96	41.74	4	80.0	1.06	0.382	ns
Week:area	382.40	95.60	4	80.0	2.43	0.055	†
Week:volume	175.02	43.76	4	80.0	1.11	0.357	ns
Week:meancurv	704.88	176.22	4	80.0	4.47	0.003	**

L temporalpole							
Week	4667.17	1166.79	4	80.0	26.13	< 0.001	***
Age	16.22	16.22	1	20.0	0.36	0.554	ns
Education	682.04	682.04	1	20.0	15.27	0.001	***
Gender	199.71	199.71	1	20.0	4.47	0.047	*
thickness	146.35	146.35	1	20.0	3.28	0.085	ns
area	204.48	204.48	1	20.0	4.58	0.045	*
volume	187.43	187.43	1	20.0	4.20	0.054	†
meancurv	907.07	907.07	1	20.0	20.31	< 0.001	***
Week:thickness	122.06	30.51	4	80.0	0.68	0.606	ns
Week:area	545.79	136.45	4	80.0	3.06	0.021	*
Week:volume	112.42	28.11	4	80.0	0.63	0.643	ns
Week:meancurv	227.26	56.81	4	80.0	1.27	0.288	ns
R parstriangularis							
Week	4564.34	1141.09	4	80.0	30.61	< 0.001	***
Age	17.12	17.12	1	20.0	0.46	0.506	ns
Education	36.83	36.83	1	20.0	0.99	0.332	ns
Gender	12.46	12.46	1	20.0	0.33	0.570	ns
thickness	1.73	1.73	1	20.0	0.05	0.832	ns
area	5.13	5.13	1	20.0	0.14	0.715	ns
volume	21.88	21.88	1	20.0	0.59	0.453	ns
meancurv	4.04	4.04	1	20.0	0.11	0.745	ns
Week:thickness	55.17	13.79	4	80.0	0.37	0.829	ns
Week:area	94.45	23.61	4	80.0	0.63	0.640	ns
Week:volume	1248.74	312.18	4	80.0	8.37	< 0.001	***
Week:meancurv	199.14	49.78	4	80.0	1.34	0.264	ns
R entorhinal							
Week	4792.35	1198.09	4	80.0	28.79	< 0.001	***
Age	183.86	183.86	1	20.0	4.42	0.048	*
Education	8.37	8.37	1	20.0	0.20	0.659	ns
Gender	1.02	1.02	1	20.0	0.02	0.877	ns
thickness	40.59	40.59	1	20.0	0.98	0.335	ns
area	11.69	11.69	1	20.0	0.28	0.602	ns
volume	251.17	251.17	1	20.0	6.04	0.023	*
meancurv	12.22	12.22	1	20.0	0.29	0.594	ns
Week:thickness	605.37	151.34	4	80.0	3.64	0.009	**
Week:area	96.25	24.06	4	80.0	0.58	0.679	ns
Week:volume	287.11	71.78	4	80.0	1.72	0.153	ns
Week:meancurv	261.51	65.38	4	80.0	1.57	0.190	ns

***, p <0.001, **, p <0.01, *, p <0.05, †, marginal, ns, non-significant.

Table S16. Cortical models for the final test performances.

Region	*AIC*	*BIC*	*logLik*	*r2m*	*r2c*	χ^2	*df*	*p (unc)*	*p (FDR)*	
baseline	675.88	697.31	-328.94	0.51	0.76					
L rostralmiddlefrontal	676.83	736.38	-313.41	0.68	0.83	31.05	16	0.013	0.048	*
L caudalmiddlefrontal	676.87	736.43	-313.44	0.70	0.82	31.00	16	0.013	0.048	*
L parsopercularis	699.76	759.31	-324.88	0.54	0.79	8.11	16	0.945	0.979	ns
L parstriangularis	674.62	734.17	-312.31	0.67	0.84	33.26	16	0.007	0.031	*
L parsorbitalis	665.77	725.32	-307.89	0.71	0.86	42.10	16	$<$ 0.001	0.006	**
L insula	681.67	741.22	-315.83	0.66	0.82	26.21	16	0.051	0.143	ns
L temporalpole	693.63	753.18	-321.81	0.59	0.80	14.25	16	0.580	0.740	ns
L bankssts	668.01	727.57	-309.01	0.69	0.86	39.86	16	0.001	0.009	**
L supramarginal	672.66	732.22	-311.33	0.70	0.84	35.21	16	0.004	0.024	*
L entorhinal	691.69	751.24	-320.85	0.63	0.79	16.18	16	0.440	0.613	ns
L parahippocampal	697.62	757.17	-323.81	0.55	0.79	10.26	16	0.853	0.953	ns
L fusiform	697.75	757.30	-323.88	0.60	0.78	10.12	16	0.860	0.953	ns
L rostralanteriorcingulate	684.54	744.09	-317.27	0.64	0.82	23.34	16	0.105	0.224	ns
L caudalanteriorcingulate	682.51	742.06	-316.26	0.70	0.80	25.36	16	0.064	0.157	ns
L precentral	673.41	732.96	-311.70	0.67	0.85	34.47	16	0.005	0.025	*
L middletemporal	690.62	750.17	-320.31	0.59	0.81	17.25	16	0.369	0.537	ns
R rostralmiddlefrontal	686.74	746.29	-318.37	0.63	0.81	21.14	16	0.173	0.292	ns
R caudalmiddlefrontal	688.08	747.63	-319.04	0.60	0.81	19.80	16	0.230	0.350	ns
R parsopercularis	701.23	760.78	-325.61	0.56	0.77	6.65	16	0.979	0.979	ns
R parstriangularis	683.89	743.44	-316.95	0.67	0.81	23.99	16	0.090	0.205	ns
R parsorbitalis	693.91	753.46	-321.96	0.60	0.79	13.96	16	0.602	0.740	ns
R insula	685.38	744.93	-317.69	0.65	0.81	22.50	16	0.128	0.241	ns
R temporalpole	699.96	759.51	-324.98	0.56	0.78	7.92	16	0.951	0.979	ns
R bankssts	685.82	745.37	-317.91	0.62	0.82	22.06	16	0.141	0.251	ns
R supramarginal	677.77	737.32	-313.89	0.65	0.84	30.10	16	0.017	0.056	†
R entorhinal	659.81	719.36	-304.91	0.75	0.86	48.06	16	$<$ 0.001	0.001	**
R parahippocampal	685.36	744.92	-317.68	0.63	0.82	22.51	16	0.127	0.241	ns
R fusiform	668.87	728.42	-309.43	0.74	0.83	39.01	16	0.001	0.009	**
R rostralanteriorcingulate	681.84	741.39	-315.92	0.67	0.81	26.04	16	0.053	0.143	ns
R caudalanteriorcingulate	687.49	747.04	-318.74	0.64	0.81	20.39	16	0.203	0.325	ns
R precentral	697.82	757.37	-323.91	0.58	0.78	10.05	16	0.864	0.953	ns
R middletemporal	692.49	752.04	-321.25	0.56	0.81	15.38	16	0.497	0.663	ns

***, p <0.001, **, p <0.01, *, p <0.05, †, marginal, ns, non-significant.

Table S17. Type III sums of squares analyses conducted on the significant cortical final test models.

Term	*Sum Sq*	*Mean Sq*	*NumDF*	*DenDF*	*F*	*p*	
R entorhinal							
TestPart	18377.41	6125.80	3	60.0	74.03	< 0.001	***
Age	412.66	412.66	1	20.0	4.99	0.037	*
Education	5.84	5.84	1	20.0	0.07	0.793	ns
Gender	80.34	80.34	1	20.0	0.97	0.336	ns
thickness	540.72	540.72	1	20.0	6.53	0.019	*
area	370.49	370.49	1	20.0	4.48	0.047	*
volume	122.87	122.87	1	20.0	1.48	0.237	ns
meancurv	773.27	773.27	1	20.0	9.35	0.006	**
TestPart:thickness	844.99	281.66	3	60.0	3.40	0.023	*
TestPart:area	119.14	39.71	3	60.0	0.48	0.697	ns
TestPart:volume	1628.88	542.96	3	60.0	6.56	0.001	***
TestPart:meancurv	1175.45	391.82	3	60.0	4.74	0.005	**
L parsorbitalis							
TestPart	18070.63	6023.54	3	60.0	70.12	< 0.001	***
Age	110.71	110.71	1	20.0	1.29	0.270	ns
Education	100.81	100.81	1	20.0	1.17	0.292	ns
Gender	102.92	102.92	1	20.0	1.20	0.287	ns
thickness	99.45	99.45	1	20.0	1.16	0.295	ns
area	13.25	13.25	1	20.0	0.15	0.699	ns
volume	854.63	854.63	1	20.0	9.95	0.005	**
meancurv	137.59	137.59	1	20.0	1.60	0.220	ns
TestPart:thickness	23.10	7.70	3	60.0	0.09	0.965	ns
TestPart:area	129.56	43.19	3	60.0	0.50	0.682	ns
TestPart:volume	3230.72	1076.91	3	60.0	12.54	< 0.001	***
TestPart:meancurv	195.95	65.32	3	60.0	0.76	0.521	ns
L bankssts							
TestPart	18210.06	6070.02	3	60.0	71.01	< 0.001	***
Age	58.12	58.12	1	20.0	0.68	0.419	ns
Education	101.01	101.01	1	20.0	1.18	0.290	ns
Gender	3.46	3.46	1	20.0	0.04	0.843	ns
thickness	591.58	591.58	1	20.0	6.92	0.016	*
area	124.58	124.58	1	20.0	1.46	0.241	ns
volume	0.11	0.11	1	20.0	0.00	0.972	ns
meancurv	198.65	198.65	1	20.0	2.32	0.143	ns
TestPart:thickness	1998.74	666.25	3	60.0	7.79	< 0.001	***
TestPart:area	971.65	323.88	3	60.0	3.79	0.015	*
TestPart:volume	139.93	46.64	3	60.0	0.55	0.653	ns
TestPart:meancurv	493.64	164.55	3	60.0	1.92	0.135	ns
R fusiform							
TestPart	17936.62	5978.87	3	60.0	59.47	< 0.001	***
Age	598.12	598.12	1	20.0	5.95	0.024	*
Education	120.50	120.50	1	20.0	1.20	0.287	ns
Gender	52.75	52.75	1	20.0	0.52	0.477	ns

thickness	2117.31	2117.31	1	20.0	21.06	< 0.001	***
area	0.55	0.55	1	20.0	0.01	0.942	ns
volume	435.96	435.96	1	20.0	4.34	0.050	ns
meancurv	1.46	1.46	1	20.0	0.01	0.905	ns
TestPart:thickness	1147.45	382.48	3	60.0	3.80	0.015	*
TestPart:area	375.51	125.17	3	60.0	1.24	0.301	ns
TestPart:volume	768.87	256.29	3	60.0	2.55	0.064	†
TestPart:meancurv	408.75	136.25	3	60.0	1.36	0.265	ns
L supramarginal							
TestPart	17901.85	5967.28	3	60.0	61.34	< 0.001	***
Age	589.61	589.61	1	20.0	6.06	0.023	*
Education	19.02	19.02	1	20.0	0.20	0.663	ns
Gender	69.28	69.28	1	20.0	0.71	0.409	ns
thickness	138.91	138.91	1	20.0	1.43	0.246	ns
area	528.14	528.14	1	20.0	5.43	0.030	*
volume	475.39	475.39	1	20.0	4.89	0.039	*
meancurv	114.75	114.75	1	20.0	1.18	0.290	ns
TestPart:thickness	500.15	166.72	3	60.0	1.71	0.174	ns
TestPart:area	1800.65	600.22	3	60.0	6.17	0.001	**
TestPart:volume	510.59	170.20	3	60.0	1.75	0.167	ns
TestPart:meancurv	84.82	28.27	3	60.0	0.29	0.832	ns
L precentral							
TestPart	18200.92	6066.97	3	60.0	65.29	< 0.001	***
Age	11.18	11.18	1	20.0	0.12	0.732	ns
Education	130.04	130.04	1	20.0	1.40	0.251	ns
Gender	117.03	117.03	1	20.0	1.26	0.275	ns
thickness	394.84	394.84	1	20.0	4.25	0.053	ns
area	36.97	36.97	1	20.0	0.40	0.535	ns
volume	12.03	12.03	1	20.0	0.13	0.723	ns
meancurv	305.08	305.08	1	20.0	3.28	0.085	ns
TestPart:thickness	1783.20	594.40	3	60.0	6.40	0.001	***
TestPart:area	372.12	124.04	3	60.0	1.33	0.271	ns
TestPart:volume	213.16	71.05	3	60.0	0.76	0.518	ns
TestPart:meancurv	789.59	263.20	3	60.0	2.83	0.046	*
L parstriangularis							
TestPart	17640.84	5880.28	3	60.0	61.65	< 0.001	***
Age	40.71	40.71	1	20.0	0.43	0.521	ns
Education	179.00	179.00	1	20.0	1.88	0.186	ns
Gender	29.41	29.41	1	20.0	0.31	0.585	ns
thickness	4.18	4.18	1	20.0	0.04	0.836	ns
area	4.49	4.49	1	20.0	0.05	0.830	ns
volume	603.90	603.90	1	20.0	6.33	0.021	*
meancurv	258.67	258.67	1	20.0	2.71	0.115	ns
TestPart:thickness	106.16	35.39	3	60.0	0.37	0.774	ns
TestPart:area	219.09	73.03	3	60.0	0.77	0.518	ns
TestPart:volume	957.70	319.23	3	60.0	3.35	0.025	*
TestPart:meancurv	1727.38	575.79	3	60.0	6.04	0.001	**

L rostralmiddlefrontal							
TestPart	17790.96	5930.32	3	60.0	59.02	< 0.001	***
Age	52.81	52.81	1	20.0	0.53	0.477	ns
Education	192.55	192.55	1	20.0	1.92	0.182	ns
Gender	301.75	301.75	1	20.0	3.00	0.098	†
thickness	7.21	7.21	1	20.0	0.07	0.792	ns
area	0.03	0.03	1	20.0	0.00	0.987	ns
volume	481.41	481.41	1	20.0	4.79	0.041	*
meancurv	448.98	448.98	1	20.0	4.47	0.047	*
TestPart:thickness	471.45	157.15	3	60.0	1.56	0.207	ns
TestPart:area	121.07	40.36	3	60.0	0.40	0.752	ns
TestPart:volume	501.27	167.09	3	60.0	1.66	0.185	ns
TestPart:meancurv	1610.53	536.84	3	60.0	5.34	0.002	**
L caudalmiddlefrontal							
TestPart	17799.60	5933.20	3	60.0	55.67	< 0.001	***
Age	418.58	418.58	1	20.0	3.93	0.061	†
Education	16.31	16.31	1	20.0	0.15	0.700	ns
Gender	483.15	483.15	1	20.0	4.53	0.046	*
thickness	0.32	0.32	1	20.0	0.00	0.957	ns
area	394.61	394.61	1	20.0	3.70	0.069	†
volume	402.58	402.58	1	20.0	3.78	0.066	†
meancurv	1066.05	1066.05	1	20.0	10.00	0.005	**
TestPart:thickness	103.77	34.59	3	60.0	0.32	0.808	ns
TestPart:area	957.30	319.10	3	60.0	2.99	0.038	*
TestPart:volume	163.89	54.63	3	60.0	0.51	0.675	ns
TestPart:meancurv	1113.51	371.17	3	60.0	3.48	0.021	*
R supramarginal							
TestPart	17098.00	5699.33	3	60.0	58.21	< 0.001	***
Age	96.91	96.91	1	20.0	0.99	0.332	ns
Education	197.04	197.04	1	20.0	2.01	0.171	ns
Gender	8.84	8.84	1	20.0	0.09	0.767	ns
thickness	26.03	26.03	1	20.0	0.27	0.612	ns
area	29.19	29.19	1	20.0	0.30	0.591	ns
volume	3.82	3.82	1	20.0	0.04	0.845	ns
meancurv	559.61	559.61	1	20.0	5.72	0.027	*
TestPart:thickness	121.03	40.34	3	60.0	0.41	0.745	ns
TestPart:area	648.52	216.17	3	60.0	2.21	0.096	†
TestPart:volume	213.94	71.31	3	60.0	0.73	0.539	ns
TestPart:meancurv	1875.45	625.15	3	60.0	6.39	0.001	***

***, p <0.001, **, p <0.01, *, p <0.05, †, marginal, ns, non-significant.

REFERENCES

Bates, E., D'Amico, S., Jacobsen, T., Székely, A., Andonova, E., Devescovi, A., et al. (2003). Timed picture naming in seven languages. *Psychonomic Bulletin & Review* 10, 344–380. doi: 10.3758/BF03196494

Chan, A. and Kwok, I. (1998). *Hong Kong List Learning Test (HKLLT): Manual and Preliminary Norms.* Hong Kong: Department of Psychology, The Chinese University of Hong Kong.

Cherry, K. E., Brown, J. S., Walker, E. J., Smitherman, E. A., Boudreaux, E. O., Volaufova, J., et al. (2012). Semantic encoding enhances the pictorial superiority effect in the oldest-old. *Aging, Neuropsychology, and Cognition* 19, 319–337. doi: 10.1080/13825585.2011.619645

Fisk, J. E. and Warr, P. (1996). Age and working memory: The role of perceptual speed, the central executive, and the phonological loop. *Psychology and Aging* 11, 316–323. doi: 10.1037/0882-7974.11.2.316

Fong, M. C.-M., Hui, N. Y., Fung, E. S.-W., Ma, M. K.-H., Law, T. S.-T., Wang, X., et al. (2020). Which cognitive functions subserve clustering and switching in category fluency? Generalisations from an extended set of semantic categories using linear mixed-effects modelling. *Quarterly Journal of Experimental Psychology* 73, 2132–2147. doi: 10.1177/1747021820957135

Fong, M. C.-M., Law, T. S. T., Ma, M. K.-H., Hui, N. Y., and Wang, W. S. (2021). Can inhibition deficit hypothesis account for age-related differences in semantic fluency? Converging evidence from Stroop color and word test and an ERP flanker task. *Brain and Language* 218, 104952. doi: 10.1016/J.BANDL.2021.104952

Goel, V. and Grafman, J. (1995). Are the frontal lobes implicated in "planning" functions? interpreting data from the Tower of Hanoi. *Neuropsychologia* 33, 623–642. doi: 10.1016/0028-3932(95)90866-P

Golden, C. and Freshwater, S. (1978). *Stroop Color and Word Test.* Chicago, IL: Stoelting.

Haarmann, H. and Usher, M. (2001). Maintenance of semantic information in capacity-limited item short-term memory. *Psychonomic Bulletin & Review* 8, 568–578. doi: 10.3758/BF03196193

Hui, N.-Y., Yuan, M., Fong, M. C.-M., and Wang, W. S. (2020). L2 proficiency predicts inhibitory ability in l1-dominant speakers. *International Journal of Bilingualism* 24, 984–998. doi: 10.1177/1367006920914399

Koda, K. (1998). The role of phonemic awareness in second language reading. *Second Language Research* 14, 194–215. doi: 10.1191/026765898676398460

Lee, T., Lo, W. K., Ching, P. C., and Meng, H. (2002). Spoken language resources for cantonese speech processing. *Speech Communication* 36, 327–342. doi: 10.1016/S0167-6393(00)00101-1

Raven, J. C. and Court, J. H. (1998). *Raven's Progressive Matrices and Vocabulary Scales.* Oxford, UK: Oxford Pyschologists Press.
